# Small molecule inhibitors targeting m^6^A regulators

**DOI:** 10.1186/s13045-024-01546-5

**Published:** 2024-05-06

**Authors:** Guotai Feng, Yongya Wu, Yuan Hu, Wen Shuai, Xiao Yang, Yong Li, Liang Ouyang, Guan Wang

**Affiliations:** 1 Department of Biotherapy, Cancer Center and State Key Laboratory of Biotherapy, Innovation Center of Nursing Research, Nursing Key Laboratory of Sichuan Province, National Clinical Research Center for Geriatrics, West China Hospital, and West China Second Hospital, Sichuan University /West China School of Nursing, Sichuan University, Chengdu, 610041 China; 2 Key Laboratory of Birth Defects and Related Diseases of Women and Children (Sichuan University), Ministry of Education, Chengdu, 610041 China

**Keywords:** N^6^-methyladenosine, Cancer therapy, Mechanism, Drug targets, Small molecule modulators

## Abstract

As the most common form of epigenetic regulation by RNA, N^6^ methyladenosine (m^6^A) modification is closely involved in physiological processes, such as growth and development, stem cell renewal and differentiation, and DNA damage response. Meanwhile, its aberrant expression in cancer tissues promotes the development of malignant tumors, as well as plays important roles in proliferation, metastasis, drug resistance, immunity and prognosis. This close association between m^6^A and cancers has garnered substantial attention in recent years. An increasing number of small molecules have emerged as potential agents to target m^6^A regulators for cancer treatment. These molecules target the epigenetic level, enabling precise intervention in RNA modifications and efficiently disrupting the survival mechanisms of tumor cells, thus paving the way for novel approaches in cancer treatment. However, there is currently a lack of a comprehensive review on small molecules targeting m^6^A regulators for anti-tumor. Here, we have comprehensively summarized the classification and functions of m^6^A regulators, elucidating their interactions with the proliferation, metastasis, drug resistance, and immune responses in common cancers. Furthermore, we have provided a comprehensive overview on the development, mode of action, pharmacology and structure–activity relationships of small molecules targeting m^6^A regulators. Our aim is to offer insights for subsequent drug design and optimization, while also providing an outlook on future prospects for small molecule development targeting m^6^A.

## Introduction

Among the modification approaches of epigenetics, methylation and demethylation modifications occupy an important position, with N^6^ methyladenosine (m^6^A) being the most common post-transcriptional RNA modification in eukaryotes [1]. As a dynamic and reversible epigenetic regulatory mechanism, m^6^A modification refers to the addition of a methyl group to the 6th nitrogen atom of adenine. It is widely expressed in RNA and mainly occurs at the RRACH sequence (R = A/G, H = A/C/U) near the stop codon and 3 '- untranslated regions (3'-UTRs). M^6^A modification is mainly regulated by three enzymes, which are Writers (such as METTL3/14) that mediate methylation, Erasers (such as FTO) that mediate demethylation, and Readers (such as YTHDF1) that recognize and bind mRNAs with m^6^A modification and thus mediate downstream reactions [2]. The three enzymes collaborate with each other to mediate RNA methylation and demethylation, regulate RNA splicing, nuclear export and degradation, and participate in RNA metabolism, thereby participating in regulating various reactions in the organism.

Compared with other targets, as an important component of epigenetic modifications, m^6^A exerts profound effects on tumors and potentially plays a role in promoting or suppressing cancer. In normal cells, m^6^A regulators not only modulate m^6^A modifications but also possess independent functions, maintaining cellular homeostasis. These cells exhibit low dependence on m^6^A regulation, capable of employing various compensatory mechanisms to counteract changes in m^6^A regulator activity, ensuring the continuity of normal functions. Conversely, in cancer cells, m^6^A regulators are often overexpressed or hyperactive, a dysregulation that disrupts normal m^6^A patterns and promotes malignancies such as tumor proliferation, survival, and invasion. The heightened dependency of cancer cells on specific m^6^A regulatory processes makes targeting m^6^A regulators an effective anticancer strategy. By affecting RNA expression and function, m^6^A modification has now been reported to be involved in proliferation, metastasis, drug resistance and immune response in a variety of cancers [3–6]. Moreover, dysregulated levels of m^6^A and its modulators have been associated with the development of cancer tissues [7]. METTL3 promotes breast cancer (BC) promotion and accelerates apoptosis by targeting the apoptosis inhibitor B-cell lymphoma-2 (Bcl-2) [8]. FTO downregulates m^6^A levels, modulates the expression of Ankyrin Repeat and SOCS Box Containing 2 (ASB2) and Retinoic Acid Receptor Alpha (RARA), enhances gene-mediated cell transformation and leukemia onset, and inhibits acute myeloid leukemia (AML) cell differentiation [9]. In summary, the treatment of tumors by targeting the regulation of m^6^A levels has already proven to be a well-established path.

METTL3 and FTO are two of the most extensively studied m^6^A modifying enzymes during tumor development. They coordinate with each other and act synergistically in a dynamically reversible m^6^A modification process. The METTL3 and FTO-related tumor signaling pathways and small molecule targeted drugs have been abundantly reported [10–16]. Additionally, there are gradual reports on the development of small molecules targeting m^6^A Readers. Currently, virtual screening, drug repurposing and rational drug design represent the primary discovery approaches for identifying small molecule compounds targeting m^6^A regulators. For example, based on the substrate analogues adenosine of the m^6^A regulator, the corresponding competitive inhibitor, such as METTL3 inhibitor UZH1a [12], was designed by virtual screening. Based on the m^6^A competition assay for the hydrophobic pocket of YTHDF1, YTHDF1 inhibitor EBSELEN [17] was designed through high-throughput screening. Based on the newly identified binding sites, spatially matched and non competitive inhibitor with micromolar IC_50_ value, such as METTL3 inhibitor CDIBA-43N [18], was screened out. Components with m^6^A inhibitory activity have been found from natural products such as FTO inhibitor Rhein [19] and METTL3 inhibitor Quercetin [11]. Through rational drug design, a series of small molecule inhibitors targeting FTO, such as FB23, FB23-2 [15], Dac51 [20], Dac85 [21], etc., were proposed through continuous optimization on inhibition activity and absorption, distribution, metabolism and extraction (ADME). Based on drug repurposing, some clinical drugs have also been found to possess m^6^A inhibitory activity, such as metformin [22]. In summary, guided by different research ideas, a large number of small molecule modulators targeting m^6^A regulators were discovered and demonstrated to have antitumor effects.

**Fig. 1:**
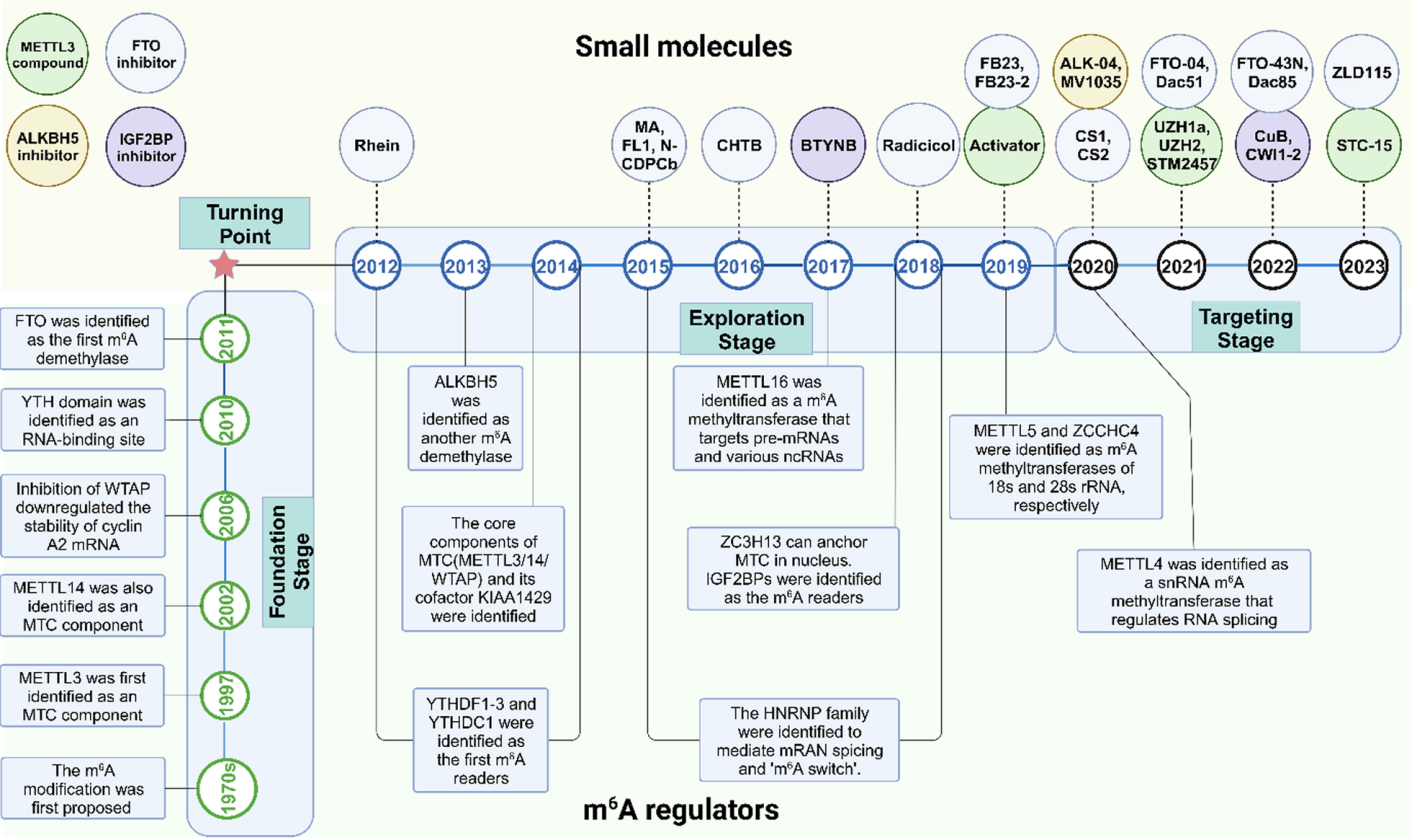
Development of m^6^A modification and small molecules targeting m^6^A regulators. Since the discovery of m^6^A in the 1970s, it was not until 1997 that the first m^6^A regulator, METTL3, was identified, and new regulators were reported over the next decade. In 2011, with the first m^6^A demethylase FTO proposed, m^6^A modification finally set up the general framework. Since 2012, researchers continued to improve the theory of m^6^A, while striving to explore and optimize small molecules targeting m^6^A. In 2023, STC-15, a derivative of the METTL3 inhibitor STM2457, became the first m^6^A target drug to enter Phase I clinical trials

Here, we present a comprehensive overview of the classification and functional roles of m^6^A regulators, shedding light on their involvement in various cancer-associated signaling pathways, elucidating their physiological significance and demonstrating the promising potential of their role as drug targets. In addition, we mainly focus on small molecules that target m^6^A regulators. We systematically categorize these molecules based on their distinct targets and design concepts, while also conducting comprehensive analysis of their structure–activity relationships (SAR), agonistic or inhibitory effects on the target, as well as their potential antitumor activity. Our ultimate goal is to provide valuable insights and directions for further optimization in designing small molecule modulators of m^6^A, thereby facilitating the discovery of more efficient and druggable m^6^A inhibitors (Fig. 1).

## m^6^A modification types

**Fig. 2:**
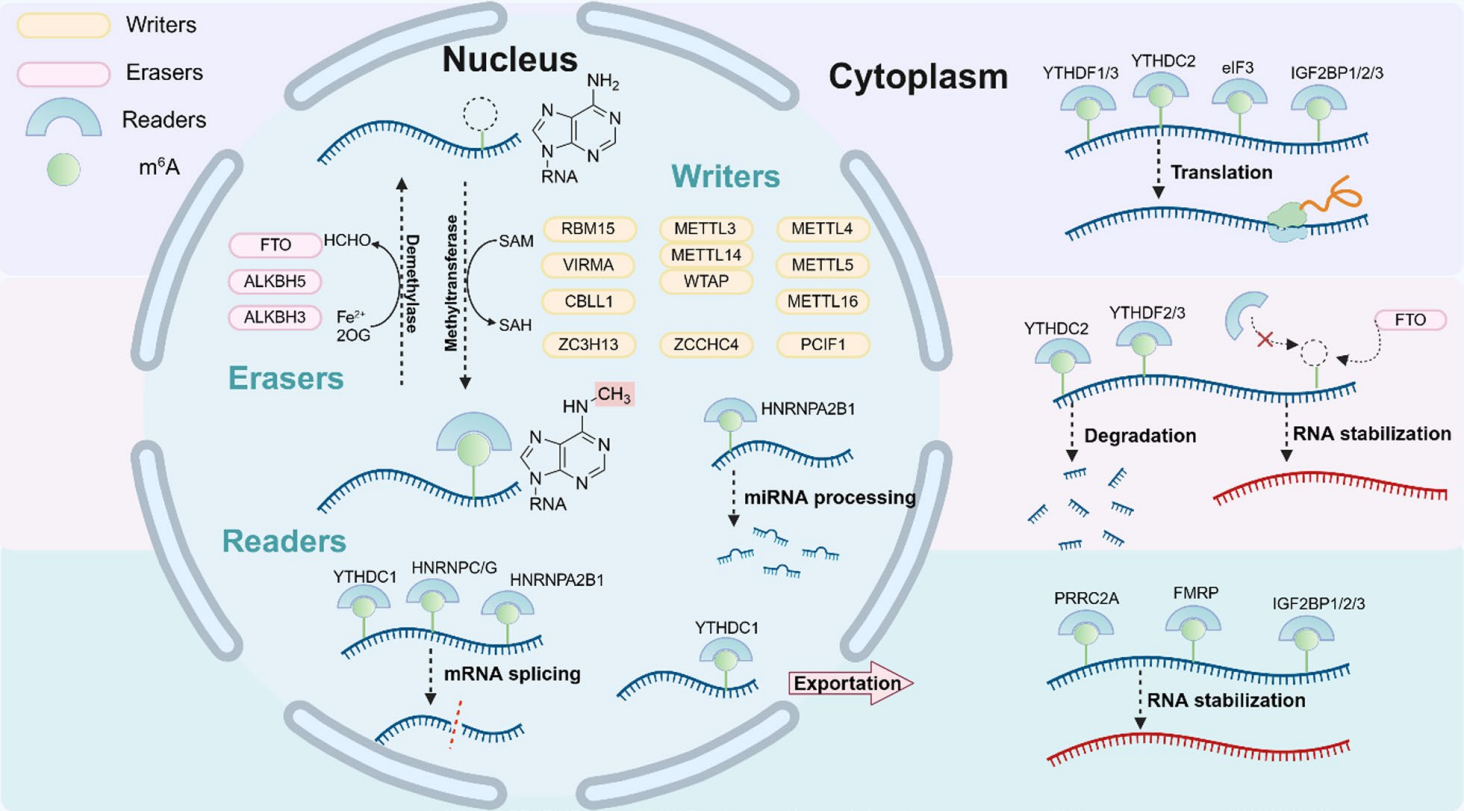
The overview of the molecular mechanism of N^6^-methyladenosine (m^6^A) modification by multiple regulators. N^6^-methyladenosine modification is a dynamic and reversible process. On the one hand, methyltransferases (Writers) such as METTL3/14/WTAP can transfer a methyl group from SAM to adenosine to complete the methylation modification; On the other hand, demethylases FTO and ALKBH5 (Erasers) can remove methylation modifications with the help of cofactors such as Fe^2+^ and 2OG. It is noteworthy that FTO can also mediate m^6^A demethylation in the cytoplasm to stabilize mRNA. Readers recognize and bind m^6^A modified RNA, allowing further processing of RNA in the nucleus and cytoplasm

M^6^A modification, as the most common post-transcriptional RNA modification, the molecular composition of its regulators mainly includes three parts, Writers, Erasers and Readers, which affect each other and collaborate with each other to regulate the methylation progress of RNA. Writers are able to convert adenosine to methyladenosine under conditions in which S-adenosylmethionine (SAM) serves as a methyl donor. Meanwhile, Erasers, with the help of their cofactors Fe^2+^ and 2OG, can mediate the demethylation modification of methyladenosine. Finally, Readers specifically bind to methylated sites and affect further processing of RNA (Fig. 2).

### Writers that mediate m^6^A methylation

The core component of the Writers is the m^6^A methyltransferase complex (MTC), which is mainly composed of three parts, METTL3, METTL4 and WTAP, and can methylate downstream RNAs to convert N^6^—adenosine to N^6^—methyladenosine. As the only catalytic component in MTC, METTL3 occupies a vital role in m^6^A modification. However, the catalytic activity of METTL3 alone is quite limited and significantly increased only when METTL3 on the nuclear speckles combines with the METTL14 in a 1:1 association to form a heterodimer complex, which activates the spatial structure of METTL3 [23, 24]. It is worth noting that METTL14, despite its lack of catalytic activity on its own, serves as an important structure for MTC. On the one hand, it can bind to METTL3 and provide significant assistance to enhance the catalytic activity of METTL3. On the other hand, it may help MTC to recognize the substrate RNA [25], facilitate RNA binding to the substrate, and enhance complex stability. In 2016, the crystal structure of the METTL3/14 methyltransferase catalytic domain complex was proposed [26], whereby the structural basis for the interaction of METTL3 with METTL14 was further elucidated. SAM is the provider of methyl groups during m^6^A methylation when catalyzing methylation reactions, and there is a cavity in the domain of METTL3 that can accommodate the methyl donor SAM. METTL3 can catalyse the transfer of a methyl group from SAM to the acceptor adenine moiety to complete RNA methylation. This process is highly coordinated with exon junction complexes (ECJs) [27]. WTAP not only serves as a stabilizer of MTC, but also structurally stabilizes it and recruits METTL3 to nuclear speckles, thereby facilitating m^6^A occurrence [28]. On the other hand, WTAP and METTL3/14 interact to affect methyltransferase activity and methylation site localization. METTL3, METTL14, and WTAP together comprise the MTC core subunits. As the core of Writers, MTC needs to localize the transcript before methylation. On the one hand, transcription factors (TFs) interact with MTCs to help their localization, thereby catalyzing m^6^A modification on transcripts in a transcription factor dependent manner [29]. On the other hand, specific histone modifications can recruit MTC to transcripts, for example, histone H3K36me3 was shown to direct the genomic localization of METTL14 in HepG2 cells [30].

In addition to MTC, other components of the Writers play important functions and are intimately involved in m^6^A modification. RBM15 / RBM15b can promote the association of METTL3/WTAP complex and the recruitment of this complex to RNA binding sites [31, 32]. VIRMA (also called KIAA1429) is involved in site-specific METTL3/14/WTAP recruitment, trafficking MTC to the 3'-UTR of mRNA with a stop codon region [31]. As an important component of methyltransferases, CBLL1 (also called HAKAI) can contact other regulators to regulate m^6^A modification [33]. Whereas ZC3H13 anchors WTAP, Virilizer, and HAKAI in the nucleus and retains MTC in nuclear speckles, enhancing its catalytic capacity [33] to facilitate m^6^A methylation. Finally, as an m^6^A methyltransferase of 28s rRNA [34], ZCCHC4 adds m^6^A to the 28s ribosomal rRNA, regulating the distribution and translation of rRNAs [35].

In recent years, some novel m^6^A regulators have also been identified. For example, METTL4 [36], which mediates U2 single stranded RNA m^6^A methylation to regulate pre mRNA splicing; METTL5 [37], which induces 18S rRNA m^6^A methylation, associates as a heterodimer with the activator tRNA methyltransferase activator subunit 11–2 (TRMT112) and shows metabolic stability; METTL16 [38, 39], which mediates m^6^A methylation of U6 single stranded RNAs (ssRNAs), non coding RNAs (ncRNAs) and pre mRNAs [40], catalyzes the m^6^A modification of the 3'-UTR of mRNAs with A43 of U^6^ small nuclear RNA (snRNA) and affects RNA stability and splicing regulation [41]; There is also the specific adenosine methyltransferase PCIF1 [42], which mediates the 3'-UTR m^6^A modification of 2'-O-methylated adenosine at the 5'-end of mRNA. As a significant part of m^6^A methylation modification, the varieties and functions of writers are still being explored and refined.

### Erasers that mediate m^6^A demethylation

M^6^A methylation modification is a dynamic and reversible process, which Erasers can use Fe^2+^ as a cofactor, α-Ketoglutarate serves as a substrate to mediate demethylation modification of RNA. During this process, FTO plays an important role with ALKBH5, the two constituent m^6^A demethylases that can oxidize the N-methyl group of m^6^A sites to hydroxymethyl (hm^6^A), formyl (f^6^A) or adenosine to achieve demethylation [43].

FTO was the first m^6^A RNA demethylase to be discovered [44]. It removes m^6^A from RNA and preferentially binds pre-mRNA in intronic regions, selectively regulating splicing and 3'-end processing [45]. In the nucleus, FTO predominantly catalyzes the demethylation of internal m^6^A. Conversely, its role in the cytoplasm extends further, not only targeting internal m^6^A and cap structure N6-2'-O-dimethyladenosine (m^6^Am) in mRNA but also acting on internal m^6^A and their cap structures in U^6^ RNA and snRNA, along with N1-methyladenosine (m^1^A) in tRNA. Moreover, FTO's cytoplasmic activity includes the catalysis of m^1^A demethylation in tRNA, which directly represses translation, highlighting its pivotal role in modulating gene expression and impacting cell functionality [43, 46].

As an important component of Erasers, ALKBH5 can regulate mRNA export and metabolism by removing methyl groups from m^6^A sites, converting m^6^A to adenine [47]. The content and distribution of ALKBH5 is extremely important for the normal expression of genes. The loss of ALKBH5 causes increased exon skipping and rapid degradation of aberrantly spliced transcripts [48]. Thus, ALKBH5 deficient male mice, develop infertility due to abnormal apoptosis of their spermatocytes. Furthermore, in cells lacking ALKBH5, the upregulation of nuclear RNA and nascent RNA significantly increases the cytoplasmic RNA levels [47]. Whereas, ALKBH5 content also varied among sites, with the highest expression in testis and lower expression in heart and brain. Various factors influence nuclear RNA yield, metabolism and gene expression [47]. Furthermore, there have been reports identifying ALKBH3 as a novel "Eraser". In contrast to FTO and ALKBH5, ALKBH3 exhibits a distinct substrate preference, focusing on tRNA rather than mRNA. Its role involves the removal of m^6^A from tRNA, and it also serves as an m^1^A demethylase in this context [49].

### Readers that bind m^6^A sites and exert specific functions

The methylation and demethylation processes of m^6^A modification are effectively facilitated through the interaction of "Writers" and "Erasers". However, for a deeper understanding of the biological role of m^6^A, the involvement of specific binding proteins, known as "Readers" is essential. These Reader proteins play a critical role by selectively recognizing m^6^A-modified sites on RNA, thereby influencing RNA translation and degradation. Examples of Reader proteins encompass the YTH family, IGF2BPs, HNRNP family, eIF3, and others.

The YTH domain family has RNA binding domains and is an important component of Readers. This family comprises two subgroups: the YTH domain family proteins 1–3, known as YTHDF1-3 (belonging to the "DF family"), and the YTH domain-containing proteins 1–2, referred to as YTHDC1-2 (part of the "DC family"). The YTHDF proteins function differently at each terminus. The YTH domain at its C-terminus helps it to bind m^6^A modified mRNA, while the N-terminal region is free to bind various cofactors for expression diversity [50]. Among them, YTHDF1 utilizes its YTH domain for the specific recognition and binding of m^6^A sites located near the mRNA stop codon. It also reassigns its N-terminus to interact with a variety of co-factors, resulting in the enhancement of translation for mRNAs containing m^6^A methylation [51]. As the first m^6^A reader to be discovered, YTHDF2 is involved in various physiological activities such as gene expression, cell death and survival [52, 53]. It has also been reported to be involved in mRNA decay, where, after binding to m^6^A methylated mRNAs, it can recruit the CCR4-NOT deadenylase complex, thereby facilitating the degradation of m^6^A-methylated mRNAs [54]. YTHDF3 serves a distinct role by interacting with different member of the YTH family. Enhances translation of methylated mRNA in cooperation with YTHDF1, and interaction with YTHDF2 accelerates mRNA decay [55, 56]. Moreover, the three are interdependent, with loss of YTHDF3 resulting in reduced binding of YTHDF1 and YTHDF2 to their target transcripts and loss of YTHDF1 or YTHDF2 also reducing the amount of RNA bound by YTHDF3 [55]. This also laterally demonstrates the close relationship of YTHDF3 with YTHDF1/2. All three collaborate with each other to jointly regulate m^6^A methylated mRNA metabolism.

In the DC family, YTHDC1 can recognize mRNAs and ncRNAs to facilitate their processing and export [57]. It achieves this by recruiting serine/arginine-rich splicing factor 3 (SRSF3) and suppressing SRSF10 to position binding sites on pre-mRNAs, thereby impacting mRNA splicing [58]. Additionally, YTHDC1 can induce the decay of various types of chromosome associated regulatory RNAs (carRNAs), encompassing promoter-associated RNAs, enhancer RNAs, and repetitive RNAs [59]. YTHDC2 is hypothesized to be a RNA helicase, consisting of YTH domains, helicase domains, R3H domains, and ankyrin repeats. It participates in the regulation of target gene translation and abundance [60]. YTHDC2 can effectively enhance translation efficiency while reducing mRNA abundance [61].

The HNRNP family is another important family protein among Readers. It consists of HNRNPA2B1, HNRNPC, and HNRNPG. The alterations of mRNA structure induced by m^6^A can enhance the binding of HNRNP proteins to m^6^A sites, which has been termed "m^6^A switches" [62–64]. HNRNPA2B1 can interact with RNA-binding protein DGCR8, a component of the pri-mRNA microprocessor complex, in an m^6^A-dependent manner to recognize primary microRNAs (pri-miRNAs). This interaction accelerates their processing, regulates splicing, and promotes mRNA maturation [65]. Additionally, HNRNPA2B1 is involved in the regulation of selective splicing of mRNA transcripts [65, 66]. HNRNP-C is an RNA-binding protein located in the cell nucleus and plays a crucial role in pre-mRNA processing [67]. HNRNP-G can recognize the ArgGly-Gly (RGG) motif in RNA low-complexity regions, selectively binding to RNA. Subsequently, it engages with the carboxy-terminal domain of RNA polymerase II (RNAPII) and m^6^A-methylated pre-mRNA to control the specific splicing of pre-mRNA [62]. In summary, HNRNP-C and HNRNP-G collaborate to process m^6^A-modified RNA transcripts, regulating mRNA abundance and splicing [62, 63].

As independent m^6^A binding proteins, IGF2BPs, including IGF2BP1, IGF2BP2 and IGF2BP3, consist of six canonical RNA binding domains, two RNA recognition motifs (RRMS) and four K-homology domains [68]. Notably, only the third and fourth KH domains (KH3-4) of the four K homology domains are essential in the recognition of m^6^A sites in mRNA. Distinct from the function of YTHDF2 in promoting mRNA decay, the presence of K homology domain can mediate IGF2BPs to recognize the consensus GG (m^6^A) C sequence, improving the translation efficiency and stability of mRNAs [69].

Additionally, recent discoveries of novel m^6^A Readers have illuminated the intricate mechanisms underlying m^6^A modification. For instance, the protein translation initiation factor eIF3 exhibits a specific affinity for m^6^A-tagged mRNAs, promoting cap-independent translation [70]. This means that m^6^A within the 5'-UTR can replace the 5'-cap structure, stimulating mRNA translation. FMRP, encoded by the fragile X mental retardation 1 gene, plays a role in augmenting the nuclear export and stability of m^6^A-modified RNA [71, 72]. Furthermore, PRRC2A, identified as a novel m^6^A Reader, selectively recognizes and binds to the GGACU motif within the oligonucleotide coding sequence, thus contributing to the stabilization of oligonucleotide mRNA [73].

In summary, m^6^A modification, one of the most vital RNA modification processes, plays a pivotal role in a wide range of biological activities, including alternative splicing, translation, and degradation. Moreover, it holds significant implications in tumor proliferation, metastasis, drug resistance, immune responses, and prognosis. A comprehensive grasp of the molecular intricacies of m^6^A modification aids in forming a more comprehensive understanding of its role in anti-tumor mechanisms (Table 1).

**Table 1:** The classification of m^6^A regulators and their functions

Type	Regulator	Full Name	Function	Ref
Writers	METTL3	Methyltransferase-like 3	Catalyzes m^6^A methylation	[23]
	METTL14	Methyltransferase-like 14	As the core subunit of MTC, forms a heterodimer with METTL3 to achieve catalytic function	[25]
	WTAP	Wilms tumor 1-associated protein	Recruits METTL3 and METTL14 to nuclear speckles and promotes m^6^A methylation	[28]
	RBM15/RBM15B	RNA-binding motif protein 15	Promotes METTL3/WTAP complex formation and binds to RNA sites	[31, 32]
	VIRMA (KIAA1429)	Vir-like m^6^A methyltransferase associated	Participates in the recruitment and transport of MTC at specific sites and mediates mRNA splicing	[31]
	CBLL1 (HAKAI)	Cbl proto-oncogene-like 1	An important component of methyltransferases	[33]
	ZC3H13	Zinc fnger CCCH-type containing 13	Retains MTC in the nuclear speck, enhances its catalytic capacity to promote m^6^A methylation	[33]
	ZCCHC4	Zinc fnger CCCH-type containing 4	Acts as an m^6^A methyltransferase of 28S rRNA to regulate its distribution and translation	[34]
	METTL4	Methyltransferase-like 4	Mediates U2 single-stranded RNA m^6^A methylation to regulate Pre-mRNA splicing	[36]
	METTL5	Methyltransferase-like 5	Induces 18S rRNA m^6^A methylation	[37]
	METTL16	Methyltransferase-like 16	Mediates the m^6^A methylation of U6 single-stranded RNA, ncRNAs and pre-mRNAs	[40]
	PCIF1	Phosphorylated CTD interacting factor 1	Mediates m^6^A modification of 2'-O-methylated adenosine at the 5'-end of mRNA	[42]
Erasers	FTO	Fat mass- and obesity- associated protein	Removes the methyl group at the m^6^A site and regulates RNA splicing	[44, 45]
	ALKBH5	ALKB homolog 5	Removes the methyl group at the m^6^A site and regulates RNA metabolism and export	[47]
	ALKBH3	ALKB homolog 3	Removes m^6^A from tRNA or act as an m^1^A demethylase	[49]
Readers	YTHDF1	YTH domain family protein 1	Promotes mRNA translation	[51]
	YTHDF2	YTH domain family protein 2	Promotes mRNA degradation	[52, 53]
	YTHDF3	YTH domain family protein 3	Interacts with YTHDF1 to promote mRNA translation or interacts with YTHDF2 to promote mRNA degradation	[55, 56]
	YTHDC1	YTH domain-containing protein 1	Promotes mRNA processing, export and splicing	[57–59]
	YTHDC2	YTH domain-containing protein 2	Improves translation efficiency and reduces mRNA abundance	[60]
	HNRNPA2B1	Heterogeneous nuclear ribonucleoproteins A2/B1	Accelerates pri-miRNAs processing, regulate splicing and promotes mRNA maturation	[65, 66]
	HNRNPC	Heterogeneous nuclear ribonucleoproteins C	Promotes pre-mRNA processing	[67]
	HNRNPG	Heterogeneous nuclear ribonucleoproteins G	Regulates alternative splicing of pre-mRNA	[62]
	eIF3	Eukaryotic translation initiation factor 3 subunit A	Promotes mRNA translation	[248]
	IGF2BP1/2/3	Insulin-like growth factor 2 mRNA-binding protein 1/2/3	Improves the efficiency and stability of mRNAs translation	[69]
	FMRP	Fragile X mental retardation protein	Promotes nuclear export and stability of m^6^A modified RNA	[71, 72]
	PRRC2A	Proline rich coiled-coil 2A	Stabilizes the oligonucleotide mRNA	[73]

## Potential of m^6^A in cancer therapy

**Fig. 3:**
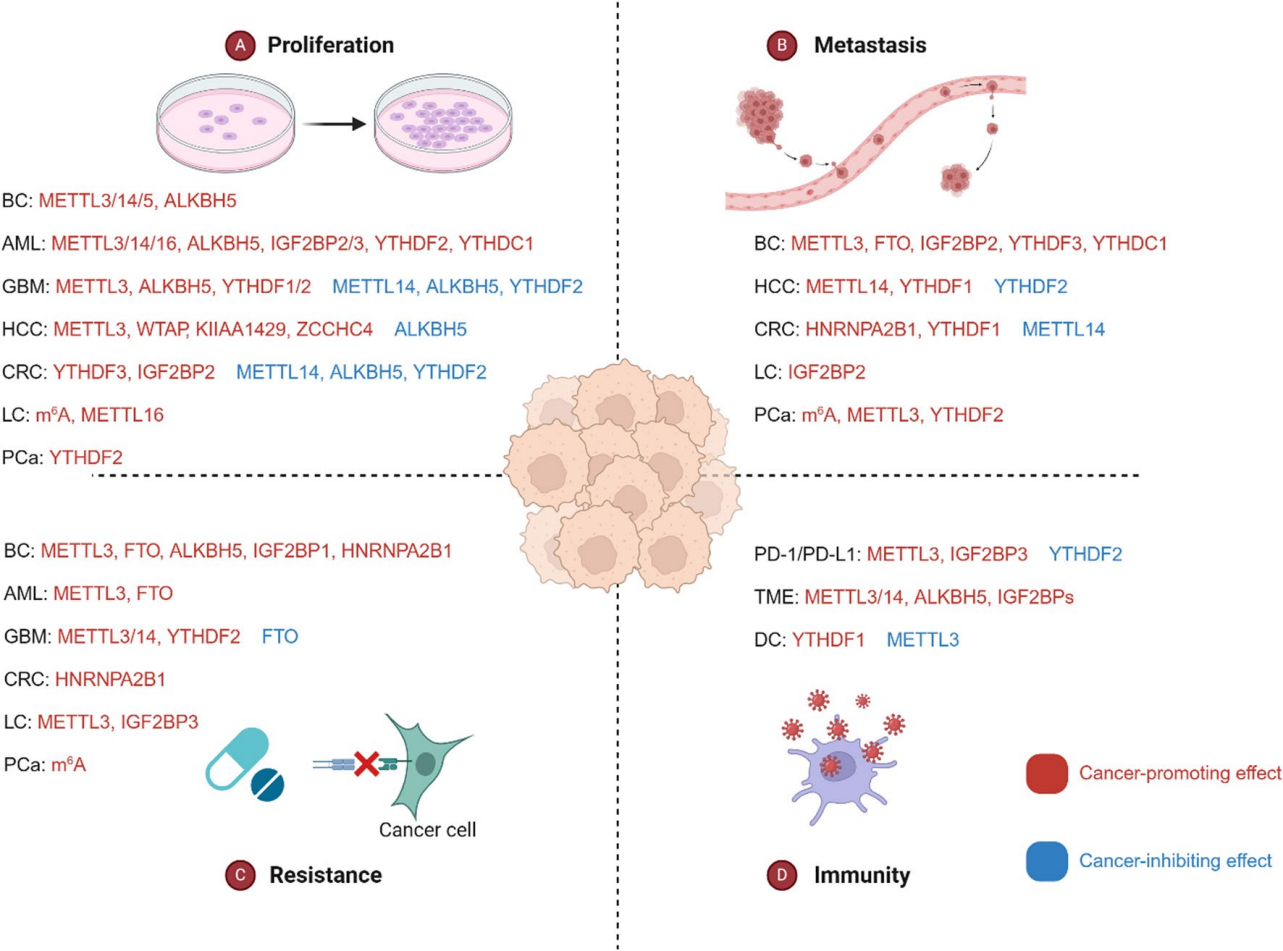
The correlation between m^6^A regulators and the processes of cancer proliferation, metastasis, resistance and immunity. M^6^A regulators can be involved in regulating the physiological activities of cancer cells through many diverse signaling pathways, and different types of cancer cell proliferation, metastasis, drug resistance, and immunity are associated with different m^6^A regulators. Where regulators of red font have a promoting effect on tumor development and regulators of blue font have an inhibitory effect on tumor development

M^6^A modification is the most common way of RNA modification, and its regulators Writers, Erasers and Readers can comprehensively regulate N^6^-methyladenosine modification from many aspects. These regulators play integral roles in numerous biological processes, notably in the context of tumor development. Therefore, to further elucidate the therapeutic potential of m^6^A modification in cancer, here we started with the understanding of proliferation, metastasis, drug resistance, immunity, the tumor microenvironment (TME) and prognosis (Fig. 3).

### m^6^A mediates tumor cell proliferation and renewal

METTL3/14 expression is elevated in many cancer tissues including BC, AML, glioblastoma (GBM) and hepatocellular carcinoma (HCC) and is closely associated with cancer cell proliferation. Studies have found that knockdown of METTL3/14 reduces methylation levels, inhibits cell proliferation, and accelerates apoptosis, leading to inhibition of tumor growth [8, 74–76]. This implies that targeting METTL3/14 is an effective therapeutic path, but the specific mechanism is more complex and remains to be further explored.

First, METTL3/14 levels of cells are subjected to an integrated regulation by multiple enzymes or regulators. Wang and colleagues conducted experiments revealing that the overexpression of the anti-apoptotic protein B-cell lymphoma-2 could counteract the apoptosis of BC cells induced by the silencing of METTL3. This observation implies that METTL3 may facilitate BC proliferation and impede apoptosis by targeting Bcl-2 [8]. Aurora kinase A (AURKA) plays a crucial role in guiding cells through mitosis. In the context of BC cell proliferation, it extensively controls the expression of RNase III DROSHA through two pathways. On the one hand, ubiquitinated degradation of m^6^A Writer METTL14 was inhibited by AURKA and overexpressed METTL14 promoted DROSHA mRNA methylation. On the other hand, AURKA can bind DROSHA transcripts, further enhancing the binding of m^6^A Reader IGF2BP2 to transcripts and stabilizing m^6^A modified DROSHA. After that, the highly expressed DROSHA interacts with β-Catenin to transactivate stemness gene STC1 in an RNA cleavage-independent manner, promoting breast cancer stem cell (BCSC) properties and mediating BC cell proliferation [77]. In the leukaemia cell line K562, elevated METTL3/14 expression was associated with increased cell proliferation. Conversely, when METTL3/14 was knocked down, it led to the inhibition of cell proliferation, induced apoptosis and differentiation [74], and brought about alterations in p53 signaling. This, in turn, resulted in the downregulation of p53, cyclin-dependent kinase inhibitor 1a (CDKN1A), and mouse double minute 2 (mdm2) [78], ultimately contributing to the delayed progression of leukemia. In conclusion, METTL3/14 serves an oncogenic role in AML by impacting the mdm2/p53 signaling pathway. METTL3 was also found to be highly expressed in GBM and associated with maintenance of Glioblastoma Stem Cells (GSCs) versus dedifferentiation, exhibiting GBM associated oncogenic effects. On the one hand, METTL3 can activate the NOTCH pathway and promote gliomagenesis by regulating the mRNA and protein levels of delta like ligand 3 (DLL3), neurogenic locus notch homolog protein 3 (NOTCH3) and hairy and enhancer of split 1 (HES1) [75]. On the other hand, downregulating METTL3 can decrease the level of m^6^A modification of SRSF and reduce its protein level, which affects alternative splicing and inhibits the proliferation and self-renewal of GSCs [79, 80]. Besides, platelet derived growth factor (PDGF) signaling can induce METTL3, decrease the tumor-suppressive Optineurin Gene (OPTN) protein levels and promote GSC proliferation and self-renewal [81]. In the context of HCC, METTL3 is capable of facilitating m^6^A methylation of the suppressor of cytokine signaling 2 (SOCS2) gene. Subsequently, YTHDF2 recognizes and binds to this m^6^A modification site, leading to the degradation of SOCS2 mRNA. This cascade of events further accelerates the development of HCC [76].

Second, METTL3 can promote tumor proliferation by regulating the methylation of various RNAs such as miRNAs, lncRNAs, and circRNAs to affect their expression. Based on the observation in clinical BC tissues that METTL3 and mammalian hepatitis B x-interacting protein (HBXIP) are positively correlated with the development of breast malignancy. Cai et al. discovered that HBXIP can elevate METTL3 levels by suppressing the BC tumor suppressor miRNA, let-7g. In turn, METTL3 increases the expression of HBXIP, creating a positive feedback loop involving HBXIP/let-7g/METTL3/HBXIP. This loop accelerates the proliferation of BC cells [82]. Sun and colleagues reported that LINC00942 (LNC942), a lncRNA possessing a specific recognition sequence (+ 176 to + 265), has the capability to directly recruit the METTL14 protein. This recruitment facilitates METTL14-mediated m^6^A methylation, which, in turn, regulates the expression and stability of C-X-C motif chemokine receptor 4 (CXCR4) and CYP1B1. Consequently, this process promotes BC cell proliferation and clonogenicity while inhibiting BC cell apoptosis [83]. Moreover, the lncRNA PSMA3-AS1 has been found to play a role in promoting the proliferation of AML cells, specifically MV4-11, and concurrently inhibiting apoptosis. Whereas METTL3 enhances the stability of lncRNA PSMA3-AS1, PSMA3-AS1 promotes AML progression by targeting miR-20a-5p/ATG16L1 pathway to regulate autophagy levels [84]. LncRNA AUCA1 can promote AML progression by affecting the stability of METTL14 and upregulating CXCR4 and CYP1B1 expression [85]. Besides, m^6^A methylation enhances the stability of lncRNA RNA Component of Mitochondrial RNA Processing Endoribonuclease (RMRP), thereby facilitating the growth and spread of non-small cell lung cancer (NSCLC). By recruiting Y-Box Binding Protein 1 (YBX1) to the Transforming Growth Factor Beta Receptor 1 (TGFBR1) promoter, RMRP activates the TGFBR1/Smad2/Smad3 signaling pathway, accelerating the proliferation and invasion of NSCLC, and increasing resistance to therapeutic interventions [86]. As a novel circRNA, low levels of circ-0001187 significantly promote AML cell proliferation in vitro and in vivo. Mechanistically, circ-0001187 reduces mRNA m^6^A modification in AML cells by enhancing the expression of E3 ubiquitin ligase RNF113A, mediating the miR-499a-5p/RNF113A/METTL3 pathway and downregulating METTL3 expression [87]. Whereas overexpression of miR-1306-5p can directly target METTL14, downregulate its expression and slow AML progression [88]. While METTL4 predominantly functions as a promoter of cancer, it exhibits contrasting behavior in colorectal cancer (CRC). In CRC, METTL4 downregulates m^6^A levels of the long non-coding RNA X-inactive specific transcript (XIST). This downregulation impedes the degradation of XIST mRNA by YTHDF2 and consequently elevates its expression [89]. As a result, METTL4 exerts an inhibitory influence on CRC cell proliferation and invasion in this context. As a stabilizer of MTC, WTAP not only promotes AML cell proliferation and differentiation [90], but also regulates the G2/M phase and promotes HCC cell proliferation by inhibiting the post-transcriptional expression of ETS proto oncogene 1 (ETS1) via mediating m^6^A modification [91].

In addition, some newly identified m^6^A Writers are also deeply involved in cancer development. METTL5 has also recently been reported to promote translation initiation and BC cell growth, playing some role in BC progression [92]. In an m^6^A dependent manner, METTL16 promotes the expression of branched chain amino acid (BCAA) transaminase 1 (BCAT1) and BCAT2 in AML, reprogramming BCAA metabolism to sustain the renewal of leukaemia stem cells [93]. Moreover, METTL16 can inhibit the binding of Eukaryotic Translation Initiation Factor 4E Family Member 2 (eIF4E2) to the mRNA 5' cap structure, thereby diminishing the repressive effect of eIF4E2 on translation. This action facilitates the recognition of the cap structure by Eukaryotic Translation Initiation Factor 4E (eIF4E), leading to an increased synthesis of specific cancer-related proteins and consequently promoting the progression of LC [94]. As a transcription factor that regulates cell differentiation and inhibits tumour proliferation, GATA binding protein 3 (GATA3) is repressed by overexpressed KIAA1429 leading to HCC cell proliferation [95]. ZCCHC4 has also been reported to be highly expressed in HCC tissues and can significantly reduce HCC tumor size after knockdown [35].

ALKBH5 and FTO act as double-edged swords, which are upregulated in BC and AML and can exert pro-oncogenic effects by regulating mRNAs with transcription factors, but FTO is tumour suppressive for GBM. Hypoxic conditions lead to the overexpression of ALKBH5, resulting in an elevation of NANOG homeobox gene (NANOG) levels. NANOG, a key factor in cancer stem cells, plays a crucial role in the initiation and dissemination of primary tumors. A follow-up trial showed that knockdown of ALKBH5 in the human BC cell line MDA-MB-231 reduced BCSC numbers and consequently significantly reduced their capacity for tumour initiation [96]. In 2020, both Wang [97] and Shen [98] reported an indispensable role for ALKBH5 in maintaining the self-renewal of AML tumor stem cells. In the latter study, ALKBH5 was also found to be targetable by a bioactive peptide, downregulated its expression, promoted the decay of MLST8 and eukaryotic translation initiation factor 4e binding protein1 (EIF4EBP1) mRNAs, and in turn inhibited AML cell proliferation [99]. Recently, the aberrantly high expression of ALKBH5 in AML was found to be associated with a transcription factor 15 (TCF15) specifically expressed in AML stem/initiating cells (LSCs/LICs). Highly expressed ALKBH5 stabilizes inosine triphosphatase (ITPA) mRNA, leading to enhanced ITPA expression that promotes AML cell proliferation [100]. In addition, with the assistance of IGF2BP1, ALKBH5 can also repress the post-transcriptional expression of Ly6/PLAUR domain-containing protein 1 (LYPD1) to suppress HCC tumor progression by mediating m^6^A demethylation [101]. Whereas in CRC, despite little ALKBH5 expression, studies have found that external introduction of high levels of ALKBH5 can effectively suppress colorectal cancer progression by inhibiting glycolysis [101].

As the first Erasers identified, FTO is also implicated in the proliferation and renewal of a variety of cancer cells. FTO enhances gene mediated cell transformation and leukemogenesis and inhibits AML cell differentiation by downregulating m^6^A levels in mRNA transcripts and regulating the expression of targets such as RARA and ASB2 [9]. In some specific subtypes of AML, such as AML with nucleophosmin 1 (NPM1) mutations, FTO promotes AML cell survival from multiple angles. On the one hand, NPM1 mutant A can promote cell cycle and inhibit apoptosis by upregulating FTO, reducing m^6^A levels, and activating the PDGFRb/extracellular signal regulated kinase (ERK) signalling axis, which in turn maintains AML cell viability [102]. On the other hand, FTO mediated m^6^A modification also upregulates the expression of the tumour protein p53 inducible nuclear protein 2 (TP53INP2) in the cytoplasm of NPM1 mutated AML cells, which further promotes leukaemia cell survival by enhancing their autophagic activity [103]. In GBM, FTO plays a distinct antitumor role. From the miRNA perspective, on the one hand, the transcription factor SPI1 can inhibit the activity of FTO, regulate the modification and processing of primary miRNA-10a (pri-miR-10a), and promote GBM progression [104]. On the other hand, miR-27a-3p, which is highly expressed in hypoxic GBM, can directly bind to and inhibit FTO expression, thereby inhibiting forkhead box O3 (FOXO3a) nuclear translocation, downregulating the expression of FOXO3a downstream target genes and inducing the malignant behavior of GBM [105].

LncRNAs, miRNAs, circRNAs, and some signaling regulators can coordinately regulate tumor cell proliferation by closely linking m^6^A modification through Readers. First, researchers identified an abnormally upregulated hypoxia-inducible lncRNA known as kb-1980e3 in clinical BC tissues. Mechanistically, lncRNA kb-1980e3 functions by recruiting IGF2BP1, forming a signaling axis involving lncRNA kb-1980e3, IGF2BP1, and c-Myc. This axis enhances the binding of IGF2BP1 to the coding region of c-Myc, leading to the stabilization of c-Myc mRNA. Consequently, this mechanism sustains the stemness and promotes self-renewal and tumorigenesis of BCSCs [106]. IGF2BP2 both improves the stability of lncRNA DANC and plays an important role in AML cell proliferation [107]. In turn, it can bind to lncRNA CASC9 and form a stable complex, thereby increasing the stability and activity of hexokinase 2 (HK2) mRNA and promoting the occurrence of aerobic glycolysis in GBM cells, which provides energy and raw materials for GBM cell proliferation [108]. Its ubiquitinated degradation can also be inhibited by circrna enhancer of zeste homolog 2 (EZH2), leading to aggravation of CRC [109] through elevated expression of the transcription factor cyclic AMP response binding protein 1 (CREB1). Under hypoxic conditions, the highly expressed long non-coding RNA STEAP3-AS1 competes with YTHDF2 for STEAP3 metalloreductase (STEAP3) mRNA, thereby shielding it from degradation. This action elevates intracellular iron levels and inhibits the activity of glycogen synthase kinase 3β (GSK3β), activating the Wnt/β-catenin signaling pathway and promoting the progression of CRC [110].

Second, as for the signal regulators, IGF2BP2 can affect the glutamine metabolism pathway by regulating the expression of key targets, such as Myc, GPT2, and solute carrier family 1 member 5 (SLC1A5), which in turn promotes AML development and LSC/LIC self-renewal 111]. Similarly, IGF2BP3 stabilizes the expression of regulator of chromosome condensation 2 (RCC2) mRNA to promote AML progression [112]. The YTH family is an important component of m^6^A readers, and both YTHDF1 [113] and YTHDF2 [114] promote AML cell proliferation by regulating m^6^A methylation. Yarmishyn [115] reported Musashi-1 (MSI1), a post-transcriptional gene expression regulator implicated in GBM, whose expression is positively correlated with YTHDF1. Interestingly, MSI1 can relieve the inhibition of GBM cell proliferation caused by low expression of YTHDF1, implying that both play an oncogenic role in GBM development. Similar to YTHDF1, YTHDF2 is also an oncogenic factor for GBM. The consistent activation of the EGFR/SRC/ERK pathway in GBM cells results in the upregulation of YTHDF2, which in turn downregulates liver X receptor A (LXRA) and hivep zinc finger 2 (HIVEP2) mRNA expression, thereby promoting GBM cell proliferation [116]. M^6^A modified inflammatory factors are also associated with tumorigenesis. MiRNA-145 [117] and HIF-2α [118] both regulate YTHDF2 levels in HCC, however low levels of YTHDF2 fail to catalyze the decay of m^6^A modified interleukin 11 (IL11) and serpin family e member 2 (SERPINE2) mRNAs, which in turn leads to the initiation of inflammation and exacerbation of malignancy in HCC. Moreover, in prostate cancer (PCa), YTHDF2 directly binds to the m^6^A modification sites of Phospholysine Phosphohistidine Inorganic Pyrophosphate Phosphatase (LHPP) and NK3 Homeobox 1 (NKX3-1), facilitating the degradation of mRNA and thereby reducing their expression levels within cells. This process activates Akt phosphorylation, inducing proliferation and migration in PCa [53]. YTHDF3 can elevate the expression of YAP, a key effector of the Hippo pathway, to play an important role in CRC development by promoting m^6^A modified lncRNA GAS5 degradation [119]. YTHDC1 is one of the most reported m^6^A binding proteins, and on the one hand, nuclear YTHDC1-m^6^A condensates (nYACs) induce AML cell survival and maintenance in an undifferentiated state [120]. On the other hand, YTHDC1 can promote AML cell proliferation and renewal by mediating MCM4 [121], a key regulator of DNA replication, and regulating the alternative splicing of HOXB-AS3 [122].

### m^6^A mediates tumor cell metastasis

During the intermediate and advanced stages of cancer, patients develop manifestations of cachexia accompanied by cancer cell metastasis, which is a significant contributor to cancer lethality. Research have found that m^6^A regulators can regulate BC cells migration and invasion through multiple signaling pathways and are closely involved in the metastatic progression of BC. METTL3 can upregulate the expression of the cancer promoting lncRNA metastasis associated lung adenocarcinoma transcript 1 (MALAT1) to promote EMT, migration and BC infiltration [123]. In general, METTL3 needs to enter the nucleus to exert its function. Its overexpression leads to an increased risk of BC metastasis through multiple pathways. It has been reported that METTL3 mediates m^6^A modification to participate in the mRNA transcription of IL-6, which would promote the deacetylation of METTL3 at K177 site, and subsequently deacetylated METTL3 is able to enter the nucleus, mediate nuclear translocation, induce global m^6^A abundance, and thus induce metastasis of BC. The combined action of aspirin and SIRT1 inhibits the acetylation of K177, which, in turn, hinders the deacetylation of METTL3. This prevents METTL3 from entering the nucleus, weakens its nuclear function, leading to a reduction in m^6^A modification. Consequently, the synthesis of proteins that are specifically involved in promoter binding and mediated by METTL3 is compromised. Through both m^6^A-dependent and independent pathways, gene transcription is inhibited, alleviating BC metastasis [124]. While in discussing the induction mechanism of METTL3 in the nucleus, keratin KRT7 may serve as a breakthrough [125]. Specifically, the upregulation of METTL3 increases the methylation of A877 on KRT7-AS. This, in turn, enhances the stability of the KRT7-AS/KRT7 mRNA double-stranded complex through the IGF2BP1/HUR complex. This increase in stability promotes the expression of KRT7 mRNA, further contributing to lung metastasis in BC^6^. For other cancers, the bacterium Fusobacterium nucleatum (F. nucleatum) [126] can downregulate METTL3 while reducing the level of m^6^A modification, inducing CRC metastasis. In the metastatic process of PCa, m^6^A modification plays a crucial role through multiple mechanisms. Firstly, it enhances the stability and expression of Nuclear Factor I B (NFIB) mRNA, activating the EMT process and driving the metastasis of castration-resistant PCa [127]. Secondly, it reduces Ubiquitin Specific Peptidase 4 (USP4) expression, leading to the degradation of ELAV Like RNA Binding Protein 1 (ELAVL1) protein and subsequently increasing Rho GDP Dissociation Inhibitor Alpha (ARHGDIA) levels, promoting cancer cell invasion and migration [128]. Thirdly, it facilitates the binding of CYCLINL1 to Cyclin-Dependent Kinase 19 (CDK19), activating RNA Polymerase II Serine 2 (Pol II Ser2) phosphorylation and increasing Runt-Related Transcription Factor 2 (RUNX2) expression, aiding in the bone metastasis of PCa [129]. These pathways collectively highlight the pivotal regulatory role of m6A in the progression of PCa, offering new targets for therapeutic intervention. Furthermore, by interacting with the microprocessor protein DGCR8, and mediating m^6^A modification of miRNA 126, METTL14 is able to suppress HCC tumour metastasis [130]. The m^6^A demethylase FTO has also been reported to regulate BC cell migration and invasion through the miR-181b-3p/ARL5B signalling pathway [131].

In triple-negative breast cancer (TNBC), TGF-β1 directly activates the Smad3 signaling pathway to regulate tissue fibrosis. Recent studies suggest that the m^6^A reader YTHDC1, which mediates mRNA processing, export, and splicing, enhances the nuclear export and expression of Smad3, thereby mediating the Transforming Growth Factor-beta 1 (TGF-β1) signaling pathway. This leads to increased survival and TGF-β1-induced EMT and promotes distant metastasis in TNBC. Targeting the YTHDC1/m^6^A/Smad3 axis for the treatment of TNBC has also emerged as a promising pathway [132]. In 2020, Chang et al. reported that overexpressed YTHDF3 could enhance m^6^A enriched brain metastasis associated transcripts such as ST6GALNAC5, GJA1 and EGFR, while there was a clinical link with BC brain metastasis [133]. Elevated expression of YTHDF1 in patients with CRC tumors is strongly linked to cancer cell metastasis. This effect is primarily mediated through the upregulation of the protein-coding gene Rho/RAC guanine nucleotide exchange factor 2 (ARHGEF2) [134]. While high expression of YTHDF1 and EGFR were also confirmed to be associated with HCC cell metastasis [135]. In addition, IGF2BP2 enhances the RNA stability of Fms Related Tyrosine Kinase 4 (FLT4) via m^6^A modification, activating the PI3K-Akt signaling pathway in lung adenocarcinoma (LUDA). This activation promotes angiogenesis and LUDA metastasis [136].

### m^6^A mediates tumor cell resistance

Currently cancer treatment is still dominated by chemotherapy and radiotherapy, but the drug resistance of tumors greatly hinders the recovery of patients. For anti-tumor drugs, m^6^A modification can affect mRNA and protein expression, leading to tumor resistance. First, m^6^A modification of RNA can affect ATP binding cassette (ABC) transporters, thereby modulating their Multidrug Resistance (MDR) [137, 138]. For example, an increase in m^6^A modification levels can trigger the splicing of estrogen-related receptor gamma gene (ESRRG) mRNA, resulting in the upregulation of estrogen-related receptor gamma (ERRγ) expression in BC drug-resistant tumor cells. This upregulation leads to increased expression of ATP-binding cassette subfamily B member 1 (ABCB1) and carnitine palmitoyltransferase 1B (CPT1B), ultimately causing chemotherapy resistance in BC cells [139].

Second, m^6^A modification also affects downstream effects in cells by regulating organismal gene expression. The study by Sun et al. demonstrates that METTL3 contributes to chemotherapy resistance in small cell lung cancer (SCLC) by enhancing the m^6^A methylation of DCP2, leading to its degradation. This activation of the Pink1-Parkin pathway subsequently promotes mitophagy, culminating in chemotherapy resistance [140]. Li et al. showed that METTL3, through methylating modifications on integrin alpha 4 (ITGA4) mRNA, increases ITGA4 protein expression and promotes homing/engraftment of AML cells, thereby mediating chemoresistance [141]. Wang also reported that the tumour suppressor genes MEG3 and miRNA-493-5p, which are lowly expressed in AML cells, upregulate the METTL3/Myc axis, thereby promoting chemoresistance in AML cells [142]. But at the same time, there are some studies that expressed the opposite point. Adipogenesis of bone marrow mesenchymal stem cells (MSCs) promotes chemoresistance in AML cells, and METTL3 can significantly inhibit MSC adipogenesis by mediating m^6^A modification to affect AKT Serine/Threonine Kinase (Akt1) mRNA and reduce Akt protein expression in MSCs [143] and improve drug resistance in AML MSCs. This implies that, in different cells, METTL3 also exhibits different roles, and both high expression of METTL3 in AML cells and low expression in MSCs promote the development of drug resistance. Furthermore, FTO was found to be overexpressed in AML relapse samples, and knockdown of FTO increased the chemosensitivity of AML cells by elevating FOXO3 [144]. Several other factors also profoundly influence tumour resistance, with senescent neutrophil derived exosomal piRNA-17560 enhancing FTO expression, and upregulation of FTO further enhances ZEB1 transcript stability and expression by reducing m^6^A RNA methylation, leading to tumour cell chemoresistance and EMT [145]. Whereas ALKBH5 promoted m^6^A demethylation of the sugar transporter GLUT4 mRNA in a YTHDF2 dependent manner, increased GLUT4 mRNA stability and promoted glycolysis in BC cells. Overexpression of ALKBH5 renders BC cells resistant to Human Epidermal Growth Factor Receptor 2 (HER2) targeted therapy [146]. As an m^6^A reader protein, IGF2BP3 recognizes m^6^A sites on Cytochrome C Oxidase Subunit 6B2 (COX6B2) mRNA, enhancing its stability and upregulating COX6B2 expression. This process facilitates metabolic reprogramming in NSCLC, leading to drug resistance [147].

For specific drugs, the resistance of common antineoplastic drugs, such as tamoxifen (TAM), adriamycin (ADR), docetaxel (DP), and so on, are all intricately linked to m^6^A modification. In TAM-resistant BC MCF-7 cells, a significant increase in the protein levels of both adenylate kinase 4 (AK4) and METTL3 is observed. Further mechanistic investigations have revealed that inhibiting METTL3 results in decreased levels of the AK4 protein. This combined effect sensitizes TAMR MCF-7 cells to TAM [148]. Interestingly, contrasting viewpoints have been put forward, suggesting that knocking down METTL3 in HR^+^/HER2^−^ BC cells can lead to the modulation of CDKN1A/EMT and m^6^A-bax/caspase-9/-3/-8 signaling pathways. In some cases, this modulation appears to promote proliferation and migration while reducing drug sensitivity [149]. This may suggest to us that METTL3 plays diametrically opposite roles in different signaling pathways or different cancer subtypes. In addition to METTL3, HNRNPA2B1 overexpression also enhanced the resistance of MCF-7 to tamoxifen and fulvestrant, suggesting that HNRNPA2B1 has a role in possible endocrine resistance [150] and that inhibition of HNRNPA2B1 might be a completely new road to treating endocrine resistant BC [151].

Doxorubicin, as a chemotherapeutic drug that can induce DNA damage, m^6^A modification to induce DNA repair to mediate tumor resistance to doxorubicin. For example, protein arginine methyltransferase 5 (PRMT5) inhibits m^6^A methylation by enhancing the nuclear translocation of ALKBH5, further enhancing DNA repair capacity and thereby reducing the efficacy of doxorubicin in BC cells [152]. Other m^6^A regulators such as METTL3 and YTHDF1 are also involved in chemoresistance [153] by mediating the EGF/RAD51 axis, enhancing homologous recombination repair (HR) to improve DNA damage repair in BC [154]. Furthermore, studies conducted by Pan [155] and Wang [156] have indicated that resistance to doxorubicin is associated with the expression of METTL3 and FTO. While METTL3 and FTO have opposing roles in the regulation of m^6^A modification, it's noteworthy that due to differences in the signaling pathways involved, genetic knockdown of either of these genes enhances the sensitivity of BC cells to doxorubicin. METTL3 has also been found to be involved in doxorubicin resistance of AML cells, and inhibition of METTL3 increased the sensitivity of drug-resistant cells and inhibited the proliferation of doxorubicin resistant HL60/ADR cells [157]. Moreover, WTAP has also been implicated in the resistance of AML cells to doxorubicin [158]. In conclusion, m^6^A regulators are closely involved in doxorubicin resistance by enhancing tumor cell DNA repair function or mediating drug-resistant cell proliferation.

Docetaxel, a taxane based chemotherapeutic, has been widely used in antitumor therapy. Overexpressed METTL3 mediates the resistance of TNBC to docetaxel, and although miR-186-5p can bind to METTL3 and inhibit its expression, in turn, METTL3 can also induce the lncRNA LINC 00662 to compete with miR-186-5p for binding to METTL3, thereby promoting its own expression [159]. Temozolomide resistance has been a major obstacle in the treatment of GBM, and significantly higher levels of METTL3 have been found in resistant GBM cells. Li's research demonstrated that temozolomide leads to an increase in histone H3K27ac levels and the recruitment of RNA polymerase II, thereby inducing an SRY box transcription factor 4 (SOX4)-mediated upregulation of METTL3. Notably, through the combined approach of temozolomide treatment and METTL3 silencing, trials showed that it was possible to inhibit the growth of temozolomide-resistant orthotopic graft tumors [160]. Shi reported further mechanism on this basis that METTL3 mediates drug resistance by modifying DNA repair genes MGMT and APNG [161], while Yin proposed that METTL3-modified lncRNA00839 is also involved in this process by activating the Wnt/β-catenin signaling pathway [162]. In conclusion, silencing METTL3 is perhaps key to improving radiotherapy resistance.

In addition to mediating METTL3 involvement in drug resistance, lncRNA located at the X-inactive specific transcript (JPX) enhance phosphatidylinositol dependent kinase 1 (PDK1 mRNA) stability in an FTO dependent form, promoting temozolomide resistance [163]. In addition, YTHDF2 also inhibits the expression of EPH receptor B3 (EPHB3) and Tumor Necrosis Factor α Induced Protein 3 (TNFAIP3) in an m^6^A-dependent manner, activating Phosphoinositide 3-Kinases (PI3K)/Akt and nuclear factor kappa-B (NF-κB) signaling, promoting resistance in GBM [164]. In addition to temozolomide, anti-angiogenic agents and mTOR inhibitors used in the treatment of GBM also face challenges of drug resistance. For angiogenesis inhibitors, vasculogenic mimicry (VM) is the biggest obstacle for its drug resistance. In GBM cells, knockdown of BUD13, cyclin dependent kinase 12 (CDK12), or overexpression of muscleblind like 1 (MBNL1) have been reported to inhibit GBM VM formation. METTL3 mediated m^6^A modification upregulated its protein expression by stabilizing BUD13 mRNA. Consequently, this stabilization enhanced CDK12 expression while suppressing MBNL1 content [165]. Targeting METTL3 to regulate the BUD13/CDK12/MBNL1 axis may provide new ideas for improving GBM resistance. For mTOR inhibitors, the translation of mRNA via the internal ribosome entry site (IRES) mechanism facilitates the synthesis of proteins that confer resistance in GBM. This IRES activation is dependent on the expression of METTL3/14, which respond to the exposure of inhibitors. GBM cell line sensitivity can be improved by silencing METTL3/14 [166]. Finally, HNRNPA2B1 can interact with the lncRNA MIR100HG to promote CRC resistance to cetuximab and metastasis by regulating TCF7L2 mRNA stability [167].

Radiotherapy mainly plays an anti-tumor role by damaging cancer cell DNA, and the resistance of GSCs to radiotherapy mainly derives from their highly efficient DNA repair ability. Visvanathan reported that because METTL3 promotes HR [168] by stabilizing SOX2, silencing METTL3 can disable the DNA repair capacity of GSCs and sensitize them to radiotherapy in the recovery phase. In addition, highly expressed ALKBH5 increases radiotherapy resistance by regulating HR. Whereas, in ALKBH5 deficient cells, the radiation sensitivity of GSCs rises owing to decreased expression of HR associated genes such as the checkpoint kinase CHK1 and RAD51 recombinase [169]. In bone metastatic PCa highly resistant to radiotherapy, research has identified that enhancer RNA (eRNA) contributes to the tumor's resistance to treatment. This is primarily due to the RNA-binding protein KH-Type Splicing Regulatory Protein (KHSRP), which recognizes m^6^A modifications on eRNA and inhibits the RNA degradation activity of the exonuclease 5'-3' Exoribonuclease 2 (XRN2) [170]. Therefore, targeting KHSRP inhibition emerges as a potential strategy to overcome radiotherapy resistance. In conclusion, drug resistance is a key factor that causes cancers to be difficult to treat and relapse, and these m^6^A modification related drug resistance studies have undoubtedly provided us with valuable new ideas to overcome tumor drug resistance.

### m^6^A mediates anti-tumor immunity, TME and prognosis

As the guardian of human health, the immune system has the functions of initiating body defense and maintaining body homeostasis. The immune checkpoint PD-1/PD-L1 is an important member of the immune circuit and is able to regulate the magnitude of immune responses and mediate tumor resistance and immune homeostasis. Meanwhile, tumors can also exploit immune checkpoints for immune escape, which promotes their own malignant development. The link between m^6^A modification and immune checkpoints can help us better understand tumorigenesis and explore more efficient therapies.

As a downstream target of m^6^A methylation modification, the immune checkpoint PD-L1 is modified by METTL3, and PD-L1 mRNA stability can be increased by METTL3 mediated m^6^A methylation. METTL3 can also upregulate PD-L1 expression at the post-transcriptional level via an IGF2BP3 dependent manner, thereby further promoting PD-L1 mRNA stabilization. PD-L1 and METTL3 expression is positively correlated, Knockdown of METTL3 enhances antitumour immunity via PD-L1-mediated T cell activation, exhaustion and infiltration [171]. When METTL3 is absent, YTHDF2 stabilizes the signal transducer and activator of transcription 1 (STAT1), leading to the augmentation of immune responses to anti-PD-1 therapy [172]. METTL3 also synergizes with YTHDF1 to promote myeloid derived suppressor cell (MDSC) migration and suppress body immunity by promoting chemokine (C-X-C motif) ligand 1 (CXCL1)/CXCR2 expression in an m^6^A dependent manner [173, 174]. Briefly, low levels of METTL3 enhance the body's immune response.

Beyond immune checkpoints, cellular immune factors, such as dendritic cells (DCs) and natural killer (NK) cells, play important roles in the body's immune response. DCs, in particular, hold essential functions in antigen presentation within both innate and adaptive immunity [175]. Studies have shown that METTL3 can promote DC activation and function, and deletion of METTL3 inhibits the antigen-presenting properties of DCS in vivo and in vitro. In terms of the mechanism, METTL3 accelerates the translation of CD40, CD80, and Tirap transcripts in DCs. At the same time, METTL3 enhances T cell activation with cytokine production [176]. The lowly expressed m^6^A binding protein ythdf1 inhibits lysosomal proteolysis in DCs, promotes cross presentation of tumor antigens, and thereby enhances PD-L1 therapeutic efficacy. Further study showed that the efficacy of combined YTHDF1 knockdown and anti-PD-L1 treatment was quite good, superior to that of single agent control group, and combining m^6^A with immune checkpoint inhibitor (ICI) treatment is promising in tumor therapy.

The TME not only underpins the foundation for tumor growth and dissemination but also exerts a critical influence on tumor progression, invasiveness, and the response to treatment through the regulation of intricate interactions between cancer cells and their surrounding milieu. A pivotal example of such regulation involves the m^6^A reader IGF2BPs, which play distinct roles in LC and BC. In LC, circular RNA NADH Dehydrogenase (Ubiquinone) 1 Beta Subcomplex Subunit 2 (circNDUFB2) effectively activates Retinoic Acid Inducible Gene I (RIG-I) by promoting the binding and accelerated ubiquitination and degradation of IGF2BPs with Tripartite Motif Containing 25 (TRIM25), recruiting immune cells to the TME. This dual mechanism significantly inhibits LC progression [177]. Conversely, in BC, IGF2BP2 binds to the lncRNA small nuclear RNA host gene 5 (SNHG5) in Cancer-Associated Fibroblasts (CAFs). This interaction notably stabilizes ZNF281 mRNA, culminating in the enhanced expression of chemokines (CCL2 and CCL5) and the activation of the P38 mitogen-activated protein kinase (MAPK) signaling pathway. These mechanisms are instrumental in promoting angiogenesis and establishing a conducive niche for the onset of BC metastasis [178]. These insights highlight the pivotal role of the TME in cancer progression and underscore the unique mechanisms of action of IGF2BPs across different cancer types, suggesting the TME as a potential target for cancer therapy. Besides, silencing METTL3/14 in TME affects the production of cytokines and chemokines, ultimately sensitizing tumor cells to interferon-γ treatment [172]. In addition to METTL3/14, ALKBH5 also increases PD-L1 expression and regulates the TME [179–182]. Unlike METTL3, knockdown of ALKBH5 increases m^6^A modification of PD-L1 mRNA, promoting PD -L1 degradation in a YTHDF2 dependent manner. In addition, ALKBH5 suppresses T cell proliferation and cytotoxicity by upregulating PD-L1 expression, effectively reducing tumor cell infiltration. Specifically, under hypoxic conditions, increased expression of ALKBH5 stabilizes lncRNA nuclear paraspeckle assembly transcript1 (NEAT1), leading to elevated CXCL8/IL8 expression, crucial for recruiting tumor-associated macrophages (TAMs). TAMs have a tumor promoting role and are involved in the formation of an immunosuppressive TME in GBM. ALKBH5 has thus been shown to mediate an immunosuppressive TME [183]. Additionally, research has shown that immune checkpoints and galectin signaling pathways, mediated by m^6^A, also facilitate the formation of an immunosuppressive TME [184]. The immunosuppressive TME presents a significant challenge for the immunotherapy of GBM, with effective solutions still lacking. To address this issue, Qiu's 2022 study [185] highlighted a novel therapeutic strategy targeting the YY1-CDK9 transcriptional elongation complex. This approach, activating interferons via m^6^A modification, reduces T cell infiltration and enhances the anti-PD-1 response, offering a new direction for GBM treatment. Moreover, the m^6^A modified pseudogene HSPA7 is a novel prognostically relevant biomarker that occupies an important role in GBM associated immune activation and oncogenic pathways. In vitro assays found that HSPA7 was positively correlated with TAM expression, and knockdown of HSPA7 increased the efficiency of anti-PD-1 therapy, implying that HSPA7 might be a novel immunotherapeutic target [186].

**Fig. 4:**
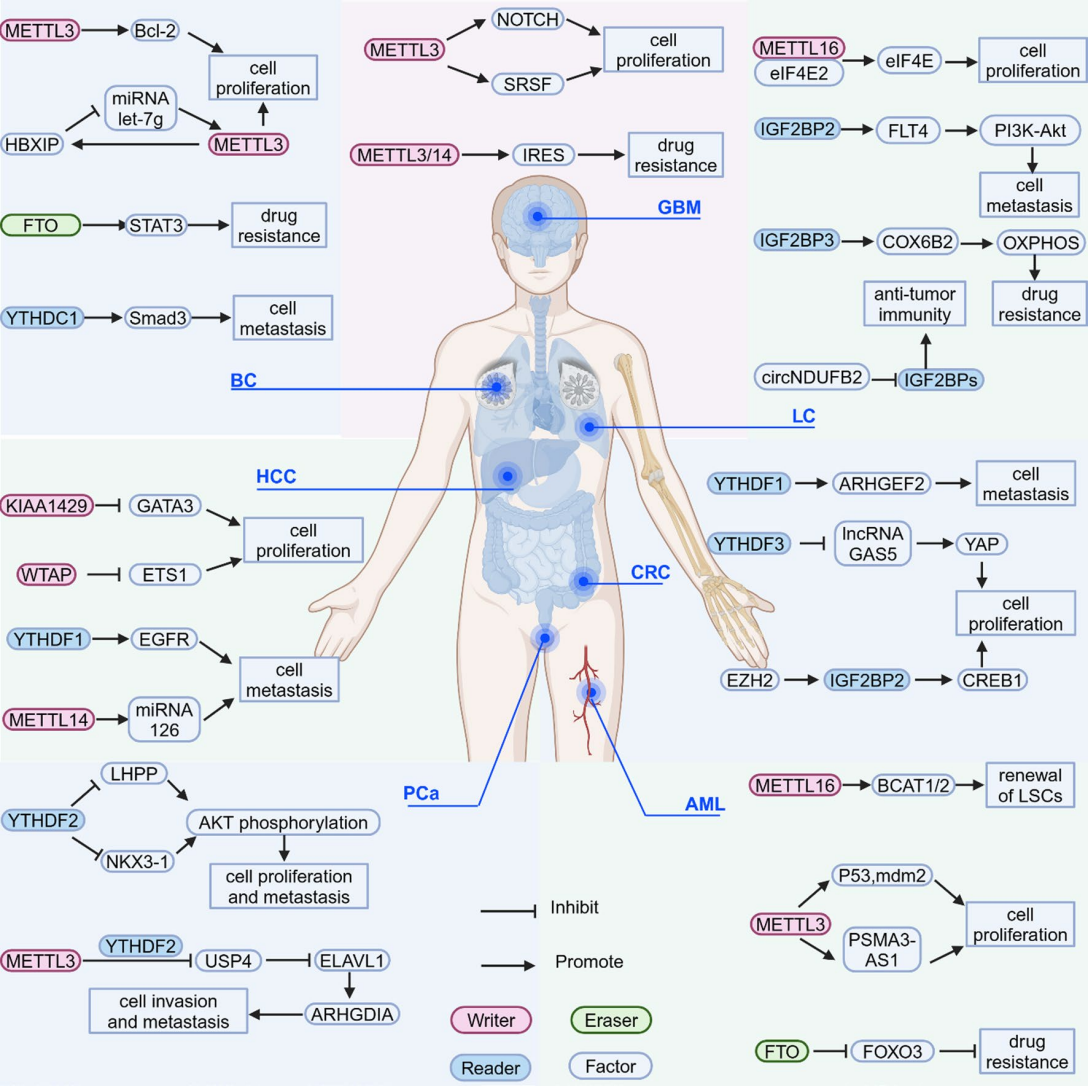
The m^6^A modification regulates cancer development by mediating different signaling pathways. For BC, GBM, AML, HCC, and CRC, m^6^A modification can promote or inhibit signaling factors to regulate the physiological activities of cancer cells. The figure illustrates some of these pathways

Numerous studies have shown a well-established approach to develop tumor prognostic models based on m^6^A modification patterns for the evaluation and guidance of immunotherapy. By conducting comprehensive analyses of a substantial cohort of AML patients with a focus on m^6^A modifications [187–189], researchers systematically examined the overlaps and clustering within differentially expressed genes (DEGs). Based on these findings, they developed a scoring system known as the m^6^A score. Patients were scored by detecting m^6^A levels in their plasma. In patients with low m^6^A scores, higher expression of immune regulators PD-L1, PD-L2, MRP1, and MRP2 was associated with higher tumor mutation and infiltration rates. While patients with high m^6^A scores not only had better 5-year survival rate, they also showed more advantages in clinical treatment. In GBM, patients with high GM score tend to be more immunocompromised than those with low GM score due to immune escape caused by T-cell dysfunction. This prompted us that improving our understanding of TME infiltration and guiding immunotherapy by assessing m^6^A patterns for tumor prognosis. Given that m^6^A modified lncRNAs have been linked to multiple pathways in cancer, this suggests that it is also a highly potential signature for tumour prognosis [190]. Recently, Yang [191] reported that there were 17 m^6^A regulators whose expression differed between AML resistant group and sensitive group with high correlation. He used m^6^A regulators to establish a prediction model of AML resistance to cytarabine, which could be used for adjuvant treatment of AML resistance. Whereas in the above mentioned srsf expression is closely related to GSC proliferation and renewal [79, 80]. In addition, it is also highly related to multiple immune regulators. Therefore, SRSF also has the potential to be a novel biomarker for immunotherapy and prognosis [192]. (Table 2, Fig. 4).

**Table 2:** The relationship between m6A modification and cancers

Cancer	Type	Regulator	Expression	Signal axis	Function	Ref
BC	Writers	METTL3	Up-regulated	HBXIP/let-7g/METTL3/HBXIP	Promotes proliferation	[82]
			Up-regulated	METTL3/Bcl-2	Promotes proliferation	[8]
			Up-regulated	METTL3/MALAT1/miR-2^6^b/HMGA2	Promotes metastasis	[123]
			Down-regulated	AMPK/SIRT1	Inhibits metastasis	[124]
			Up-regulated	METTL3/IGF2BP1-HUR complex/KRT7 AS-KRT7 mRNA ds/KRT7 mRNA	Promotes metastasis	[6]
			Up-regulated	METTL3/ESRRG mRNA/ERRγ/ABCB1 and CPT1B	Promotes drug resistance	[139]
			Up-regulated	METTL3/AK4/ROS and P38	Promotes drug resistance	[148]
			Up-regulated	EGF/RAD51	Promotes drug resistance	[154]
			Up-regulated	METTL3/miR-221-3p/HIPK2/Che-1	Promotes drug resistance	[155]
			Up-regulated	METTL3/LINC00662/miR-186-5p	Promotes drug resistance	[159]
			Down-regulated	CDKN1A/EMT m^6^A-bax/caspase-9/-3/-8	Reduces drug resistance	[149]
			Up-regulated	IFN-γ/STAT1/Irf1	Renders the tumor insensitive to PD inhibition	[103]
		METTL14	Up-regulated	AURKA/METTL14/IGF2BP2/DROSHA/STC1	Promotes proliferation	[77]
			Up-regulated	LNC942/METTL14/CXCR4,CYP1B1	Promotes proliferation	[83]
		METTL5	Up-regulated	METTL5/p70-S6K	Promotes proliferation	[92]
	Erasers	FTO	Up-regulated	FTO/STAT3	Promotes drug resistance	[156]
			Up-regulated	FTO/miR-181b-3p/ARL5B	Promotes metastasis	[131]
		ALKBH5	Up-regulated	ALKBH5/NANOG mRNA/NANOG	Promotes proliferation	[96]
			Up-regulated	PRMT5/ALKBH5/m^6^A/DNA repair	Promotes drug resistance	[152]
			Up-regulated	ALKBH5/GLUT4 mRNA	Promotes drug resistance	[146]
	Readers	IGF2BP1	Up-regulated	lncRNA kb-1980e3/IGF2BP1/c-Myc	Promotes drug resistance	[106]
		IGF2BP2	Up-regulated	LncSNHG5/IGF2BP2/ZNF281 mRNA/ CCL2 and CCL5/P38 MAPK signaling	Promotes metastasis	[178]
		YTHDF3	Up-regulated	YTHDF3/ST6GALNAC5, GJA1 and EGFR transcripts	Promotes metastasis	[133]
		YTHDC1	Up-regulated	YTHDC1/m^6^A/Smad3	Promotes metastasis	[132]
		HNRNPA2B1	Up-regulated	/	Promotes drug resistance	[150]
AML	Writers	METTL3	Up-regulated	mdm2/p53	Promotes proliferation	[78]
			Up-regulated	METTL3/PSMA3-AS1/miR-20a-5p/ATG16L1	Promotes proliferation	[84]
			Up-regulated	Circ-0001187/miR-499a-5p/RNF113A/METTL3	Promotes proliferation	[87]
			Down-regulated	METTL3/Akt1-mRNA/Akt protein/MSC	Improves the drug resistance	[143]
			Up-regulated	METTL3/ITGA4 mRNA/ITGA4 protein	Promotes the drug resistance	[141]
			Up-regulated	MEG3/miRNA-493-5p/METTL3/MYC	Promotes the drug resistance	[142]
		METTL14	Up-regulated	lncRN AUCA1/METTL14/ CXCR4 and CYP1B1	Promotes proliferation	[85]
			Up-regulated	miR-1306-5p/METTL14	Promotes proliferation	[88]
		METTL16	Up-regulated	METTL16/m^6^A/BCAT1-2/BCAA	Maintainss the renewal of LSCs	[93]
	Erasers	FTO	Up-regulated	FTO/PDGFRB/ERK	Promotes AML cell survival	[102]
			Up-regulated	FTO/m^6^A/ TP53INP2	Promotes autophagy	[103]
			Up-regulated	FTO/m^6^A/ FOXO3	Promotes the drug resistance	[144]
		ALKBH5	Up-regulated	KDM4C/ALKBH5/AXL	Maintains the renewal of LSCs	[97]
			Up-regulated	ALKBH5/m^6^A/TACC3	Maintains the renewal of LSCs	[98]
			Up-regulated	BP/ALKBH5/ MLST8 and EIF4EBP1	Promotes proliferation	[99]
			Up-regulated	TCF15/ALKBH5/ITPA	Promotes proliferation	[100]
	Readers	IGF2BP2	Up-regulated	IGF2BP2/glutamine metabolism	Maintains the renewal of LSCs	[111]
			Up-regulated	IGF2BP2/lncRNA DANC	Promotes proliferation	[107]
		IGF2BP3	Up-regulated	IGF2BP3/RCC2 mRNA	Promotes proliferation	[112]
		YTHDF2	Up-regulated	AML1-ETO/HIF1α/YTHDF2	Promotes proliferation	[114]
		YTHDC1	Up-regulated	YTHDC1/ nYACs	Maintains an undifferentiated state	[120]
			Up-regulated	YTHDC1/ MCM4	Promotes proliferation	[121]
			Up-regulated	YTHDC1/ HOXB-AS3	Maintains the renewal of LSCs	[122]
GBM	Writers	METTL3	Up-regulated	METTL3/SRSF	Promotes proliferation	[79]
			Up-regulated	METTL3/NOTCH	Promotes proliferation	[75]
			Up-regulated	PDGF/METTL3/OPTN	Promotes proliferation	[81]
			Up-regulated	METTL3/SOX2/HR	Promotes the drug resistance	[168]
			Up-regulated	SOX4/EZH2/METTL3	Promotes the drug resistance	[160]
			Up-regulated	METTL3/MGMT and APNG	Promotes the drug resistance	[161]
			Up-regulated	METTL3/BUD13/CDK12/MBNL1	Promotes the drug resistance	[165]
		METTL3/14	Up-regulated	METTL3/14/IRES	Promotes the drug resistance	[166]
	Erasers	FTO	Down-regulated	lncRNA/FTO/PDK1	Promotes the drug resistance	[163]
			Down-regulated	SPI1/FTO/ pri-miR-10a	Inhibits proliferation	[104]
			Down-regulated	miR-27a-3p/FTO/FOXO3a	Inhibits proliferation	[105]
		ALKBH5	Up-regulated	ALKBH5/ CHK1 and RAD51/HR	Promotes radioresistance	[169]
	Readers	IGF2BP1	Up-regulated	IGF2BP2/ lncRNA CASC9	Promotes aerobic glycolysis	[108]
		YTHDF1	Up-regulated	MSI1/YTHDF1	Promotes proliferation	[115]
		YTHDF2	Up-regulated	EGFR/SRC/ERK/YTHDF2/ LXRA and HIVEP2	Promotes proliferation	[116]
			Up-regulated	YTHDF2/EPHB3 and TNFAIP3/PI3K-Akt and NF-κB	Promotes the drug resistance	[164]
HCC	Writers	METTL3	Up-regulated	METTL3/SOCS2/YTHDF2	Promotes proliferation	[76]
		METTL14	Down-regulated	METTL14/ DGCR8/miRNA 126	Inhibits metastasis	[130]
		WTAP	Up-regulated	WTAP/ ETS1	Promotes proliferation	[91]
		KIAA1429	Up-regulated	KIAA1429/ GATA3	Promotes proliferation	[95]
		ZCCHC4	Up-regulated	/	Promotes proliferation	[35]
	Erasers	ALKBH5	Down-regulated	ALKBH5/ LYPD1/IGF2BP1	Inhibits proliferation	[249]
	Readers	YTHDF1	Up-regulated	HIF-1α/YTHDF1/ATG2A, ATG14	Promotes autophagy and autophagy-related malignancy	[250]
			Up-regulated	YTHDF1/EGFR	Promotes metastasis	[135]
		YTHDF2	Down-regulated	MiRNA 145/YTHDF2	Inhibits malignancy	[117]
			Down-regulated	HIF-2α/YTHDF2/IL11, SERPINE2	Inhibits proliferation, vasculature remodeling and metastasis	[118]
CRC	Writer	METTL3	Down-regulated	F. nucleatum/METTL3/m^6^A	Inhibits metastasis	[126]
			Up-regulated	METTL3/YTHDF1/CXCL1/CXCR2	Inhibits antitumor immunity	[173, 174]
		METTL14	Down-regulated	METTL14/YTHDF2/lncRNA XIST	Inhibits proliferation and metastasis	[89]
	Erasers	ALKBH5	Down-regulated	ALKBH5/JMJD8/PKM2	Inhibits progression	[101]
	Readers	YTHDF1	Up-regulated	YTHDF1/ARHGEF2	Promotes metastasis	[134]
		YTHDF2	Down-regulated	lncRNA STEAP3-AS1/YTHDF2/Wnt/β-catenin	Inhibits progression	[110]
		YTHDF3	Up-regulated	YTHDF3/YAP	Promotes progression	[119]
		HNRNPA2B1	Up-regulated	HNRNPA2B1/lncRNA MIR100HG	Promotes the drug resistance and metastasis	[167]
		IGF2BP2	Up-regulated	circRNA EZH2/IGF2BP2	Promotes progression	[109]
LC		M^6^A	Up-regulated	m^6^A/RMRP /TGFBR1/SMAD2/SMAD3	Promotes progression	[86]
	Writers	METTL3	Up-regulated	METTL3/DCP2/Pink1-Parkin	Promotes the drug resistance	[140]
		METTL16	Up-regulated	METTL16/eIF4E2/eIF4E	Promotes progression	[94]
	Readers	IGF2BP2	Up-regulated	IGF2BP2/FLT4/PI3K/Akt	Promotes angiogenesis and metastasis	[136]
		IGF2BP3	Up-regulated	IGF2BP3/COX6B2/metabolic reprogramming	Promotes the drug resistance	[147]
		IGF2BPs	Up-regulated	circNDUFB2/IGF2BPs/RIG-I	Inhibit anti-tumor immune response	[177]
PCa		m^6^A	Up-regulated	m^6^A/NFIB/EMT	Promotes EMT and metastasis	[127]
			Up-regulated	m^6^A/NEAT1-1/phosphorylation/RUNX2	Promotes bone metastasis	[129]
			Up-regulated	KHSRP/m^6^A/eRNA	Promotes resistance to radiotherapy in bone metastatic PCa	[170]
	Writers	METTL3	Up-regulated	METTL3/USP4/ELAVL1/ARHGDIA	Promotes invasion and migration	[128]
	Readers	YTHDF2	Up-regulated	YTHDF2/LHPP/Akt	Promotes progression and metastasis	[53]

## Small-molecule compounds targeting m^6^A regulators

Accumulating evidence indicates that a strong association between the level of m^6^A modification and the occurrence and development of tumours [193]. Therefore, targeting m^6^A key proteins by small molecules and regulating their expression may bring new hope for the treatment of tumours, which is of great interest to the scientific community. Over the past decade, with the efforts of researchers, new small molecules targeting m^6^A regulators are continuously being screened and discovered, and they have shown good antitumor efficacy in vitro and in vivo. Based on targeting the different types of m^6^A modification, small molecule regulators can be classified into three categories: regulators targeting Writers, targeting Erasers, and targeting Readers. Among them, small molecules targeting Writers and Erasers account for the vast majority. As follows, we address small molecules targeting m^6^A modification in terms of their discovery, activity, structure–activity relationships, and mechanism of action (Table 3).

**Table 3:** The small molecule compounds targeting m^6^A regulators

Type	Regulator	Name	Cancer type	Structure	IC_50_/EC_50_	Ref
Writers	METTL3/14/WTAP Activitor	**1**	GBM		0.117 μM	[194]
	METTL3-14 Inhibitor	**2**	/		8..7 μM	[196]
		**3**	/		98 μM	[196]
		**4** (STM2457)	AML		16.9 nM	[10]
		**5** (STC-15)	AML		/	[197]
		**6** (UZH1a)	/		0.28 μM	[12]
		**10** (UZH2)	/		5 nM	[198]
		**11** (Quercetin)	/		2.73 μM	[11]
		**13** (CDIBA-43n)	AML		2.81 μM	[18]
		**14** (Metformin)	BC		/	[22]
		**15** (Eltrombopag)	AML		3.65 μM	[204]
Erasers	ALKBH5 Inhibitor	**16**	AML, BC		0.84 μM	[206, 251]
		**17**	AML, BC		1.79 μM	[206, 251]
		**18** (ALK-04)	Melanoma	/	/	[207]
		**19** (MV1035)	GBM		2.48 μM	[208]
		**20** (DDO-2728)	AML		2.97 μM	[209]
	FTO inhibitor	**21** (Rhein)	/		30 μM	[19]
		**22** (Clausine E)	/		27.8 μM	[212]
		**23** (Diacerein)	/		1.51 μM	[213]
		**24** (MO-I-500)	BC		8.7 μM	[5, 215, 252]
		**25**	Hela cell		0.81 μM	[216]
		**26** (MA)	HeLa cell, PCa, uterine cervical cancer		17.4 μM	[217]
		**27** (FL1)	/		6.6 μM	[219]
		**28** (N-CDPCb)	/		4.95 μM	[220]
		**29** (CHTB)	/		39.24 μM	[221]
		**30** (Radicicol)	AML, EOC		/	[222]
		**31** (FB23)	AML		0.06 μM	[15]
		**32** (FB23-2)	AML		2.6 μM	[15]
		**33** (Dac51)	SKCM, lung cancer		0.4 μM	[20]
		**34** (13a/Dac85)	AML		0.7 μM	[21]
		**35** (44/ZLD115)	AML		2.3 μM	[223]
		**36** (FTO-02)	GBM		2.18 μM	[224]
		**37** (FTO-04)	GBM		3.39 μM	[224]
		**38** (FTO-43 N)	AML, GBM		1.0 μM	[13]
		**39** (R-2HG)	AML		133.3 μM	[16]
		**40** (CS1)	AML		0.143 μM	[226]
		**41** (CS2)	AML		0.713 μM	[226]
		**43** (18,077)	BC		1.43 μM	[14]
		**44** (18,097)	BC		0.64 μM	[14]
		**45** (Entacapone)	HCC		3.5 μM	[227]
		**46** (Nafamostat mesilate)	/		13.8 μM	[228]
Readers	YTHDF1 Inhibitor	**47** (EBSELEN)	/		3.565 μM	[17]
		**48** (Tegaserod)	AML		13.82 μM	[113]
	YTHDF2 inhibitor	**49** (DC-Y13)	/		74.6 ± 1.9 μM	[235]
		**50** (DC-Y13-27)	COAD, Melanoma		21.8 ± 1.8 μM	[235]
	IGF2BP1 Inhibitor	**51** (BTYNB)	OV, Melanoma		5 μM	[236]
		**52** (CuB)	HCC		1.7 μM	[238]
	IGF2BP2 Inhibitor	**53** (JX5)	T-ALL		/	[239]
		**54** (CWI1-2)	AML		0.2 μM	[111]

### Targeting writers

#### METTL3/14/WTAP activator

**Fig. 5:**
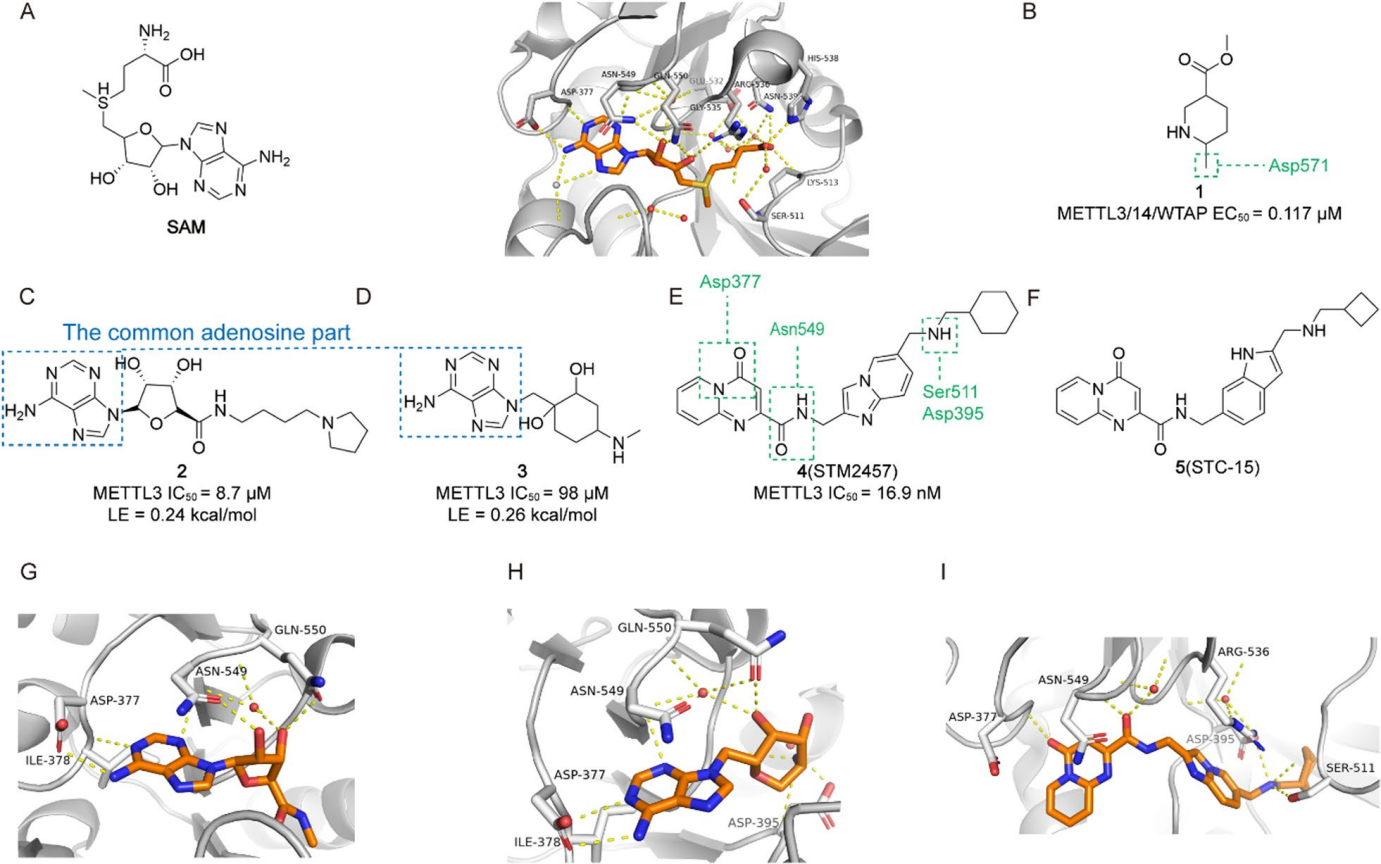
**A** Chemical structure of SAM and the crystal structure of SAM-bound METTL3/14 complex (PDB ID: 5IL1). **B** Chemical structure of METTL3/14/WTAP Activator compound **1**. **C** Chemical structure of METTL3 inhibitor compound **2**. **D** Chemical structure of METTL3 inhibitor compound **3**. **E** Chemical structure of METTL3 inhibitor compound **4**. **F** Chemical structure of METTL3 inhibitor compound **5**. **G** The crystal structure of METTL3/14 in complex with compound **2** (PDB ID: 6TTT). **H** The crystal structure of METTL3/14 in complex with compound **3** (PDB ID: 6TU1). **I** The crystal structure of METTL3/14 in complex with compound **4** (PDB ID: 7O2I)

Research has revealed that the overexpression of METTL3 inhibits the growth, self-renewal, and tumorigenesis of GSCs. Consequently, METTL3/14/WTAP activators may hold promise as potential anticancer agents against GBM [194]. In 2019, Selberg employed an in silico-based discovery approach to identify small molecule ligands that bind to METTL3/14/WTAP. Based on the conformation of protein residues connected to the SAM tail hydrogen bond, Selberg discovered a series of compounds containing piperidine and piperazine rings through virtual screening, which exhibited exceptionally high docking efficiency. Ultimately, compound **1** (methyl 6-methylpiperidine-3-carboxylate, METTL3/14/WTAP K_D_ = 47.9 pM, K_a_ = 1.58 × 10^5^ M^−1^s^−1^, Kd = 7.57 × 10^–6^ s^−1^, EC_50_ = 0.117 µM) (Fig. 5B) was identified as METTL3/14/WTAP activators. The methyl group at the para position of the ester group in compound **1** can not only strongly bind to Asp571, but also participate in hydrogen bonding with ionic interactions. Furthermore, in HEK293 cell experiments, compound **1** was able to activate METTL3/14/WTAP, resulting in a substantial increase of m^6^A mRNA by more than 40%. As a potential METTL3/14/WTAP activator leader, compound **1** can upregulate m^6^A levels, and subsequently over-expressed METTL3 can promote DNA repair and rescue UV induced DNA damage [195]. M^6^A modification plays a pivotal role in gene regulation. While considerable research has focused on inhibitors of m^6^A regulators, reports on agonists are relatively scarce. This gap highlights a potential direction for future research: the development and exploration of m^6^A regulator agonists, offering novel insights for therapeutic strategies.

#### METTL3/14 inhibitors

Substrate analogues as competitive inhibitors is a common design idea. SAM is the methyl donor to m^6^A Writers METTL3, and one of its fragments, adenosine (the other is L-methionine), is a SAM competitive inhibitor of METTL3 (IC_50_ = 500 µM). In 2020, targeting the SAM binding site of METTL3, Bedi et al. performed the drug design of METTL3 inhibitor based on adenosine. They screened 4,000 analogs and derivatives of the SAM adenosine moiety using high-throughput docking. Through in silico modeling and protein crystallographic validation, compound **2** ((2R,3S,4R,5S)-5-(6-amino-9H-purin-9-yl)-3,4-dihydroxy-N-(4-(pyrrolidin-1-yl)butyl)tetrahydrofuran-2-carboxamide, METTL3 IC_50_ = 8.7 μM, LE = 0.24 kcal/mol) (Fig. 5C) was found to exhibit good inhibitory activity, whereas compound **3** (1-((6-amino-9H-purin-9-yl)methyl)-4-(methylamino)cyclohexane-1,2-diol, METTL3 IC_50_ = 98 μM, LE = 0.26 kcal/mol) (Fig. 5D), despite its poor inhibitory activity, showed more excellent ligand binding. Both of compounds **2** and **3** exhibited significantly enhanced inhibitory activity compared with adenosine. Therefore, it is possible to draw on the structure of compound **3** to improve ligand efficiency on the basis of retaining the inhibitory activity of compound **2** and further aid the development of chemical probes fot METTL3 [196].

In 2021, based on high-throughput screening of 250,000 different drug-like compounds, Yankova et al. reported a METTL3 competitive inhibitior **4** (STM2457) [10] (Fig. 5E) with extremely strong inhibitory activity (METTL3 IC_50_ = 16.9 nM) and weak inhibitory activity against other kinases. In contrast to SAM and other METTL3 inhibitors, compound **4** establishes a unique interaction with the METTL3/14 complex through the hydrogen bondings of its two amide groups with the terminal amino groups Asp377 and Asn549 (PDB ID: 7O2I). This distinctive binding mode avoids association with the SAM homocysteine binding pocket and the conformational rearrangement of K513, Consequently, compound 4 exhibits outstanding selectivity for METTL3. Simultaneously, compound **4** cound bind to the METTL3/14 heterodimer, ultimately exerting potent inhibitory activity. In MOLM-13 cells, compound **4** was able to act at the translational level, that is, inhibiting protein expression of METTL3 biomarkers SP1 and BRD4 without affecting mRNA content. And it could effectively inhibit AML cell proliferation and clonogenicity, and induce AML cell apoptosis. Moreover, compound **4** has a sufficiently long half-life further improves its the pharmacogenic potential. STORM further developed oral-avaliable compound **5** (STC-15) [197] based on compound **4**, and is currently in phase I clinical trials. Compound **5** can inhibit METTL3/14 to exert immunomodulatory effects, and mediates changes in interferon signaling and synergistically blocks T-cell checkpoints. Furthermore, this compound showed potent efficacy in leukemia models.

**Fig. 6:**
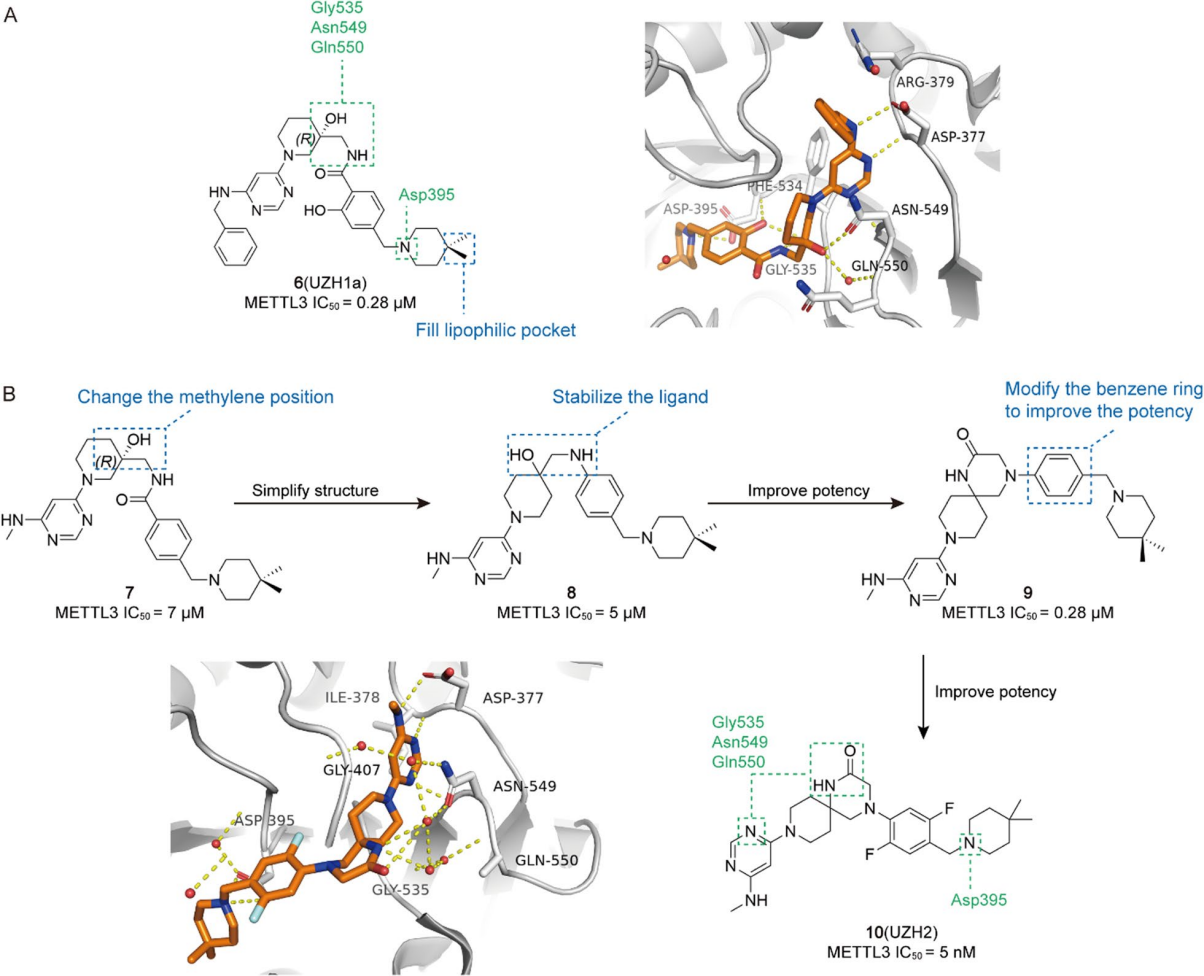
**A** Chemical structure of METTL3 inhibitor compound **6** and the crystal structure of METTL3/14 in complex with compound **6** (PDB ID: 7ACD). **B** Design and optimization process of METTL3 inhibitors compound **10** from compound **7** and the crystal structure of METTL3/14 in complex with compound **10** (PDB ID: 7O2F)

In the same year, Moroz Omori et al. performed drug design and structural optimization based on an adenine library based screen to report a selective and cell permeable METTL3 competitive inhibitor **6** (UZH1a, METTL3 IC_50_ = 0.28 μM)^12^ (Fig. 6A). The co-crystal structure reveals that compound **6** forms hydrogen bonds with the polar groups of METTL3 and occupies the binding pocket of the SAM adenosine moiety. First, the hydrogen bonds between compound **6** amide NH and phenolic oxygen are involved in the interactions between residues Gly535, Asn549 and Gln550, collectively stabilizing the compound **6**/METTL3/14 complex. Second, the tertiary amine of compound **6**, replacing the primary-amine of Lys513 in the salt bridge with Asp395, leads to a conformational rearrangement of METTL3 when bound to SAM. Finally, the dimethyl group on the piperidine fills the lipophilic pocket constituted by the residue. The various intra—and intermolecular interactions underlie the selectivity of compound **6** for METTL3. Additionally, it is necessary to pay attention to the effects of hydrogen bonding, conformational rearrangements, and pocket binding sites on METTL3 inhibitor design. Compound **6** was able to downregulate m^6^A levels of AML MOLM-13 cells (m^6^A IC_50_ = 7 μM) and osteosarcoma U2OS cells (m^6^A IC_50_ = 9 μM) by inhibiting METTL3, demonstrating its potential antitumor efficacy. Dolbois [198] had previously obtained an METTL3 inhibitor **7**((R)-4-((4,4-dimethylpiperidin-1-yl)methyl)-N-((3-hydroxy-1-(6-(methylamino)pyrimidin-4-yl)piperidin-3-yl)methyl)benzamide) by screening an adenine library, which showed similar structure to compound **6**. To simplify the structure, the investigator eliminated the carbonyl group and changed the piperidine methylene position to para, thus obtain compound **8**(4-(((4-((4,4-dimethylpiperidin-1-yl)methyl)phenyl)amino)methyl)-1-(6-(methylamino)pyrimidin-4-yl)piperidin-4-ol). Furthermore, the spirocyclic structures can make up a rigid structure, and constructing compound **9**(4-(4-((4,4-dimethylpiperidin-1-yl)methyl)phenyl)-9-(6-(methylamino)pyrimidin-4-yl)-1,4,9-triazaspiro[5.5]undecan-2-one) is beneficial to lock the ligand inside the conformation and improve the binding energy. To enhance the ADME properties of the inhibitor, Dolbois [198] explored various aspects, including the spirocyclic structure, pyrimidine, and side chains. It was found that adding a fluorine atom to the phenyl ring effectively improved the inhibitor's cellular permeability while maintaining good inhibitory activity. This improvement is attributed to the hydrophobic effect of the fluorine atom and its interaction with the nitrogen π-system. Eventually, a potent METTL3 selective inhibitor compound **10** (UZH2, METTL3 IC_50_ = 5 nM) (Fig. 6B) was obtained by structural modification and optimization of compound **7** against METTL3. Furthermore, compound **10** has certain inhibitory activity in the MOLM-13 cell line (EC_50_ = 0.7 μM) for AML and the PC-3 cell line (EC_50_ = 2.5 μM) for PCa, which suggests that the anti-tumor effect of compound **10** needs to be further developed.

**Fig. 7:**
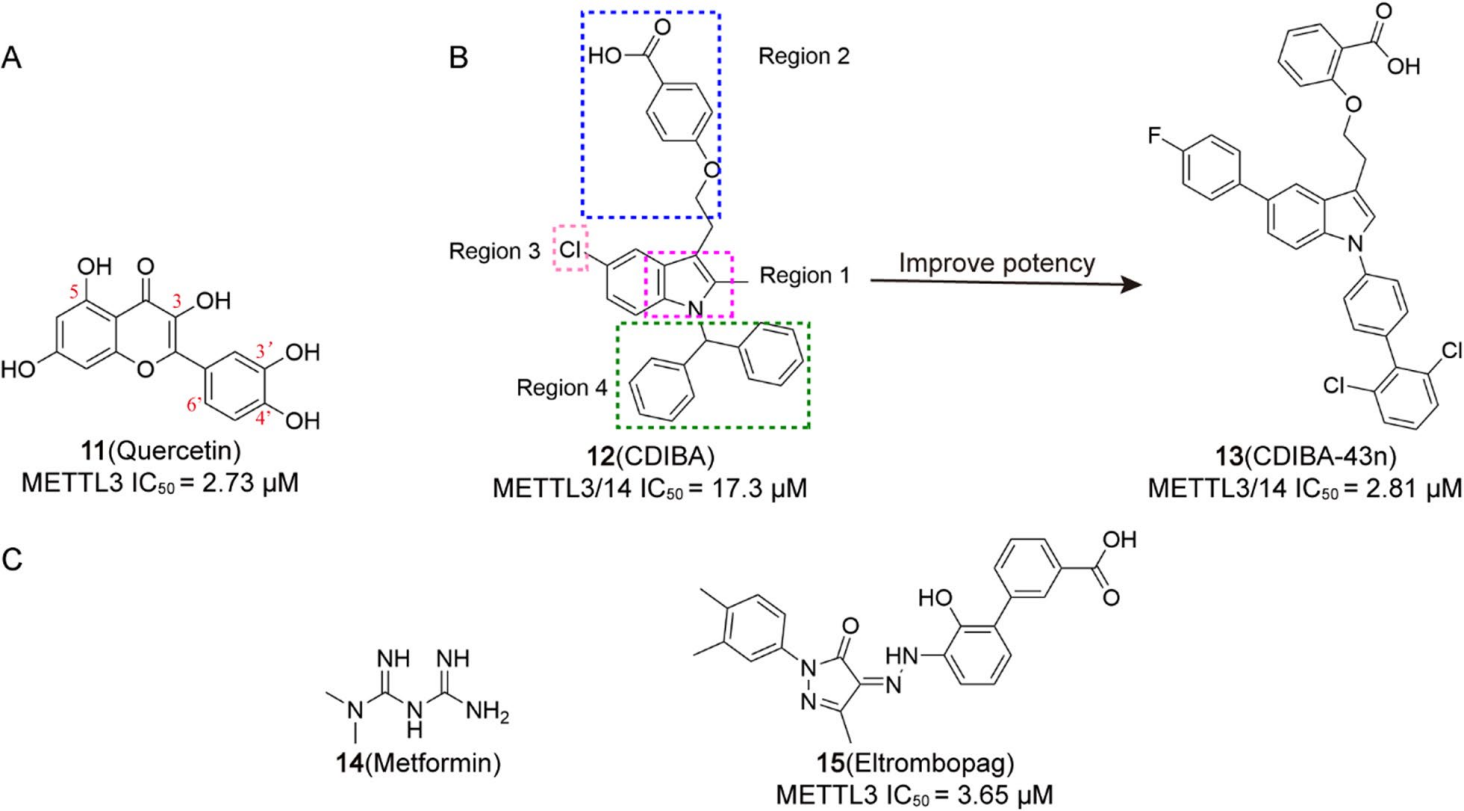
**A** Chemical structure of METTL3 inhibitor compound **11** from the natural product. **B** Design and optimization process of METTL3 inhibitor compound **13** from compound **12**. **C** Chemical structures of METTL3 inhibitors compound **14** and **15** based on drug repurposing

Natural products is a major source of new drug development. Du's group analyzed 1,042 natural products using docking-based high-throughput screening and finally identified the first natural product Quercetin (**11**) as METTL3 inhibitor (METTL3 IC_50_ = 2.73 μM)^11^ (Fig. 7A). Compound **11** could inhibit the proliferation of pancreatic cancer MIA-PaCa-2 cells (IC_50_ = 73.51 ± 11.22 Μm) and liver cancer Huh7 cells (99.97 ± 7.03 μM) by inhibiting METTL3 activity and downregulating m^6^A levels of cellular mRNAs. As one of the common natural polyphenols, compound **11** has a well-known safety profile, which also provides a basis for further structural optimization based on compound **11**, enhancing METTL3 inhibitory activity, and exploring stronger antitumor activity.

Drug repurposing has garnered significant interest in the field of drug development, as those compounds that have undergone rigorous clinical testing often demonstrate notable safety advantages when compared to newly developed drugs. Recently, there are some drugs that are already on the market, in addition to their exsiting indications, also play some regulatory role in the m^6^A field and have the potential to be targeted drugs. As SAM binding regions are contained in most methyltransferase families, the selectivity of SAM based competitive inhibitors designed can be compromised by non selective inhibitors of the methyltransferase family [199–201]. So, it is necessary to explore non-competitive inhibitors. Compound **12** (CDIBA) is a cytosolic phospholipase A2 (cPLA2) inhibitor that treats inflammation, but has also recently been reported to have METTL3/14 inhibitory activity (METTL3/14 IC_50_ = 18.3 μM) [18]. The structure of compound **12** can be divided into four regions. By individually optimizing the structures of these four regions, researcher arrived at the final noncompetitive allosteric inhibitor compound **13** (CDIBA-43n, METTL3/14 IC_50_ = 2.81 μM) (Fig. 7B) of METTL3/14. As a reversible and noncompetitive allosteric inhibitor, compound **13** was able to inhibit proliferation of a variety of AML cells (MOLM-13 cell GI_50_ = 14.6 μM, MOLM-14 cell GI_50_ = 13.1 μM, THP-1 cell GI_50_ = 21.6 μM, HL60 cell GI_50_ = 15.5 μM). Mechanistically, compound **13** downregulates m^6^A levels by targeting a region of the METTL3/14 complex that is distant from the catalytic site. Although its IC_50_ is relatively lower for conventional inhibitors that target the SAM binding site, it is far more potent and selective than for conventional inhibitors.

As a first-line treatment for type 2 diabetes mellitus (TDM2), compound **14** (metformin) (Fig. 7C) has received much attention due to its good pharmacological efficacy and convenient method for oral use [202]. As early as 2005, studies reported the therapeutic potential of compound **14** with respect to BC [203], but the specific molecular mechanisms remain unclear. In 2021, Cheng reported that the BC therapeutic potential of compound **14** may be associated with m^6^A modification. Compound **14** downregulates METTL3 by affecting miR-483-3p, reduces m^6^A methylation levels, regulates the expression of p21, and finally inhibits BC cell proliferation. In animal experiments, mice inoculated with SUM-1315 cells overexpressing METTL3 exhibited faster tumor growth and higher tumor weight compared to the control group. Meanwhile, rescue experiments indicated that metformin significantly reduced tumor weight and size [22]. Thus, compound **14** can be regarded as a METTL3 inhibitor. In 2008, the FDA approved the thrombopoietin receptor (TPO-R) agonist compound **15** (Eltrombopag, METTL3 IC_50_ = 3.65 μM) (Fig. 7C) for the treatment of chronic immune thrombocytopenia (ITP). In a recent study, compound **15** was reported as a noncompetitive allosteric inhibitor of METTL3/14 [204]. By targeting the METTL3 subunit, compound **15** selectively binds to the METTL3/14 complex and interacts with an allosteric site on the complex, affecting enzyme activity and thereby reducing m^6^A levels in cells, ultimately exhibiting activity against AML.

By designing SAM analogs and conformational inhibitors of METTL3/14, small molecules can downregulate METTL3, thereby reducing m^6^A levels, and exert anti-tumor effects. Subsequently, it may be possible to develop more potent METTL3 inhibitors by further optimizing the binding model of these compounds.

### Targeting erasers

#### ALKBH5 inhibitors

**Fig. 8:**
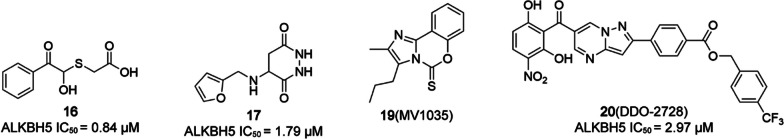
Chemical structures of ALKBH5 inhibitors compound **16** to **20**

The ALKBH5 gene has been confirmed to be a pro oncogene and regulates cancer initiation and progression by mediating multiple signaling pathways. Designing ALKBH5 inhibitors to downregulate ALKBH5 expression is a promising therapeutic avenue. In 2014, Feng [205] described the crystal structure of ALKBH5, revealing the basis of its substrate recognition and laying the groundwork for drug design targeting ALKBH5. In 2020, Simona Selberg's team used high-throughput screening and virtual screening to identify 144,000 preselected compounds and identified ALKBH5 selective inhibitors compound **16** (2-((1-hydroxy-2-oxo-2-phenylethyl)thio)acetic acid, ALKBH5 IC_50_ = 0.84 μM, LE = 0.44 kcal/mol) and compound **17** (4-((furan-2-ylmethyl)amino)tetrahydropyridazine-3,6-dione, ALKBH5 IC_50_ = 1.79 μM, LE = 0.32 kcal/mol) [206] (Fig. 8). In cell assays, low concentrations of compound **16** and compound **17** exhibited inhibitory effects on leukemia cell lines (Compound **16**, HL-60 IC_50_ = 11.9 μM, CCRF-CEM IC_50_ = 1.38 μM, K562 IC_50_ = 16.5 μM. Compound **17**, HL-60 IC_50_ = 11.0 μM, CCRF-CEM IC_50_ = 7.62 μM, K562 IC_50_ = 1.41 μM). The finding that loss of the m^6^A demethylase ALKBH5 in melanoma cells confers increased cellular sensitivity to immunotherapy [207]. This suggests that the design of ALKBH5 inhibitors may be a means to enhance the effectiveness of immunotherapy. Li's team [207] identified a small molecule ALKBH5 inhibitor, compound **18** (ALK-04), based on the X-ray crystal structure of the ALKBH5 protein through an in silico screen of compounds. Further trials found that compound **18** and GVAX/antiPD-1 immunotherapy could synergistically inhibit the growth of melanoma tumors in mice. While testing the effects of the compounds on GBM cells U87-MG found that compound **19** (MV1035) [208] (Fig. 8) expressed inhibitory activity, further investigation found that compound **19** exerted antitumor effects by inhibiting m^6^A demethylase ALKBH5 activity. Mechanistically, compound **19** could compete with 2-oxoglutarate for the binding site on ALKBH5, which is an obligatory factor for ALKBH5 to exert catalytic activity. Compound **19** does not exhibit inhibitory activity on the proliferation of GBM cells. However, by binding to the catalytic site of ALKBH5, compound **19** competitively downregulates the expression of ALKBH5, inducing a decrease in CD73 expression, and reducing the invasion and migration of GBM cells U87-MG. Recently, Wang reported a novel 2-OG-independent ALKBH5 selective inhibitor, compound **20** (DDO-2728, ALKBH5 IC_50_ = 2.97 μM) [209]. Mechanistically, compound **20** binds to ALKBH5 by occupying the m^6^A-binding pocket, exerting anti-AML cell proliferation effects through modulation of the cell cycle, E2F targets, G2M checkpoint, and MYC targets (MOLM-13 IC_50_ = 0.49 μM, MV4-11 IC_50_ = 1.2 μM). (Fig. 8).

#### FTO inhibitors

FTO, as one of the first m^6^A regulators to be discovered and studied, holds more in-depth mechanistic studies and more diverse inhibitors. In 2010, the crystal structure of a complex of FTO with mononucleotide 3-MET was reported [210], and a subsequent study found that the main active sites of FTO could be divided into 2OG binding site, Fe^2+^ and substrate binding site [211]. These reports contribute to the understanding of the substrate specificity of FTO and provide a foundation for the design of FTO inhibitors.

**Fig. 9:**
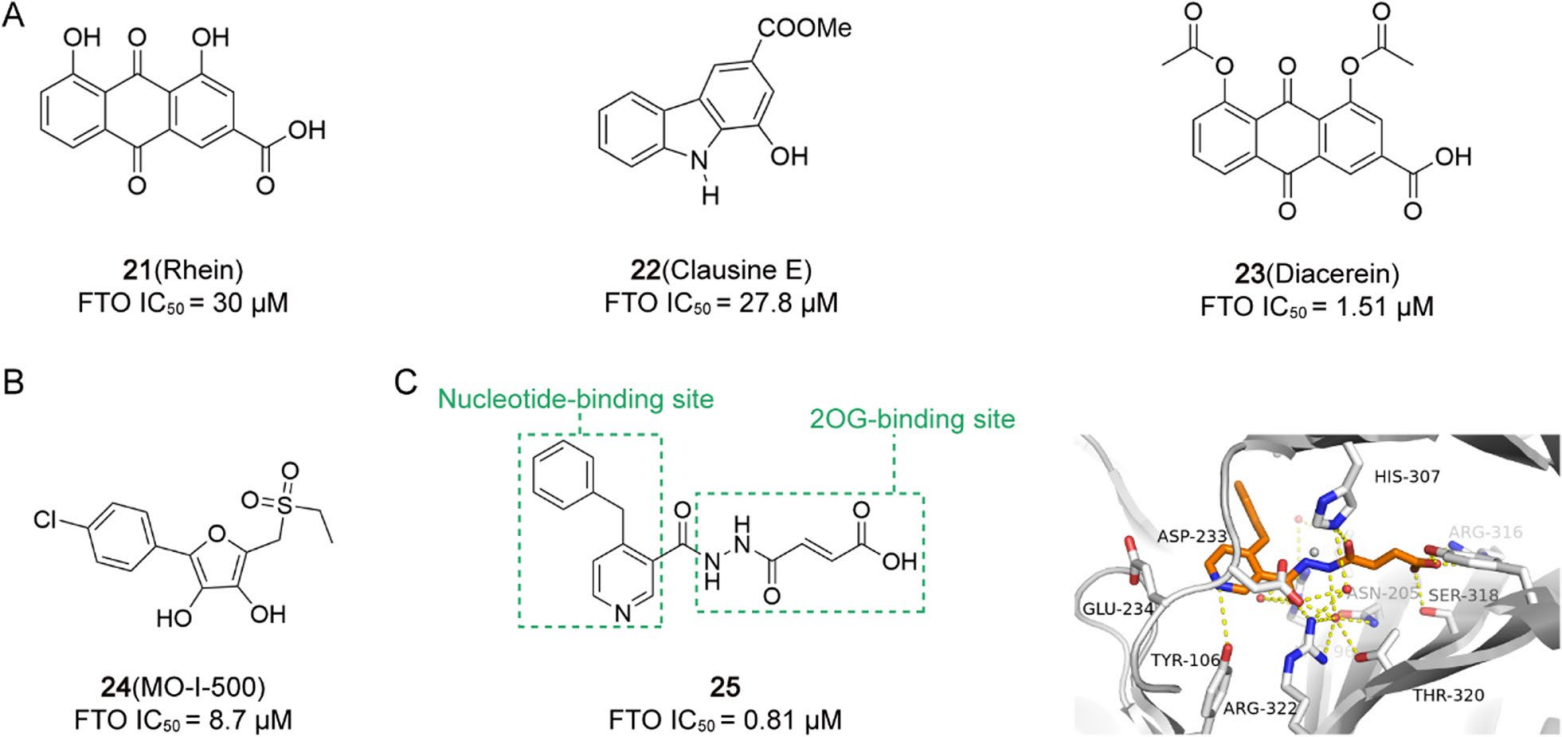
**A** Chemical structures of FTO inhibitors compounds **21–23** from natural products. **B** Chemical structure of FTO inhibitor compound **24** based on design. **C** Chemical structure of FTO inhibitor compound **25**, and the crystal structure of FTO in complex with compound **25** (PDB ID: 4CXW)

In 2012, Chen [19] identified the natural product compound **21** (Rhein, FTO IC_50_ = 30 μM, Ki = 1.8 μM) (Fig. 9A) based on virtual screening. As the first potent FTO inhibitor, it prevents FTO from binding to single stranded RNA substrates by competitively binding to the FTO catalytic domain. For the clonal cells BE(2)-C against the human neuroblastoma cell line SK-N-BE(2), only slightly molar amounts of compound **21** were able to significantly increase the m^6^A levels in BE(2)-C cells. As the first FTO inhibitor, compound **21** inevitably also suffers from drawbacks such as low inhibitory activity and weak selectivity (e.g., inability to achieve selective inhibition at ALKB family members), which need to be further improved in subsequent studies. Wang [212] also identified a new FTO inhibitor from natural products, compound **22** (Clausine E, FTO IC_50_ = 27.8 μM) (Fig. 9A). As a dose-dependent FTO inhibitor, compound **22** can use the hydroxyl and ester groups on the structure to bind into the hydrophobic cavity of FTO to exert anti-tumor activity. Zhang [213] developed a single quantum dot (QD)—based fluorescence resonance energy transfer (FRET) sensor to rapidly identify screening FTO inhibitors by detecting the decrease of Cy5 counts. With this facile tool, they found that compound **23** (Diacerein, FTO IC_50_ = 1.51 μM) (Fig. 9A), a structural analogue of compound **21**, is a specific FTO inhibitor of cellular activity. Mechanistic studies found that compound **23** exerts an inhibitory activity by competing with FTO for ssDNA. Other studies have shown that compound **23** can induce apoptosis of BC cells by mediating the interleukin-6 (IL-6) signaling pathway and exert anti-BC effects [214].

In 2014, Zheng [215] originally planned to synthesize a proline-4 hydroxylase (PHD) inhibitor but unexpectedly designed an FTO inhibitor compound **24** (MO-I-500, FTO IC_50_ = 8.7 μM) (Fig. 9B). Compound **24** exerts its therapeutic effect by chelating with the active site Fe^2+^ of FTO. Later, Singh [5] showed in the assay that, in addition to its anticonvulsant effect, compound **24** could also significantly inhibit the survival and proliferation of the drug-resistant TNBC cell line SUM149-MA by inhibiting FTO. SUM149-MA is a SUM149 TNBC cell that can survive in glutamine deprived conditions, and this ability may be associated with its high expression of FTO. In 2015, Joel D.Toh's group [216] revealed the intrinsic structural differences in the nucleotide binding site of the ALKB enzyme. They designed a 'two-component' inhibitor compound **25** ((E)-4-(2-(4-benzylnicotinoyl)hydrazineyl)-4-oxobut-2-enoic acid)(Fig. 9C) of FTO, which occupied both the 2OG and nucleotide binding sites (FTO IC_50_ = 0.81 μM), a selective FTO inhibitor with cellular activity. Crystallographic studies revealed that fumarate hydrazide 2 of compound **25** binds to the 2OG site and 4-benzylpyridine inserts into the nucleotide binding site. The carbonyl group on the compound with hydrazine can form hydrogen bonds and salt bridges with Asp233, the pyridyl ring is sandwiched between residues to constitute P-P interactions, and the 4-benzyl substituent makes hydrophobic interactions with residues. Further tests revealed that Glu234 may be the key residue determining the affinity and specificity of FTO for its substrate. Ultimately, compound **25** was identified as a potent FTO selective inhibitor through activity checks and selectivity confirmation in multiple trials. Against Hela cells, compound **25** significantly increased m^6^A levels by inhibiting FTO without affecting cellular activity. Therefore, continuing structural optimization based on key crystal sites may lead to the development of inhibitors with anti-tumor activity.

**Fig. 10:**
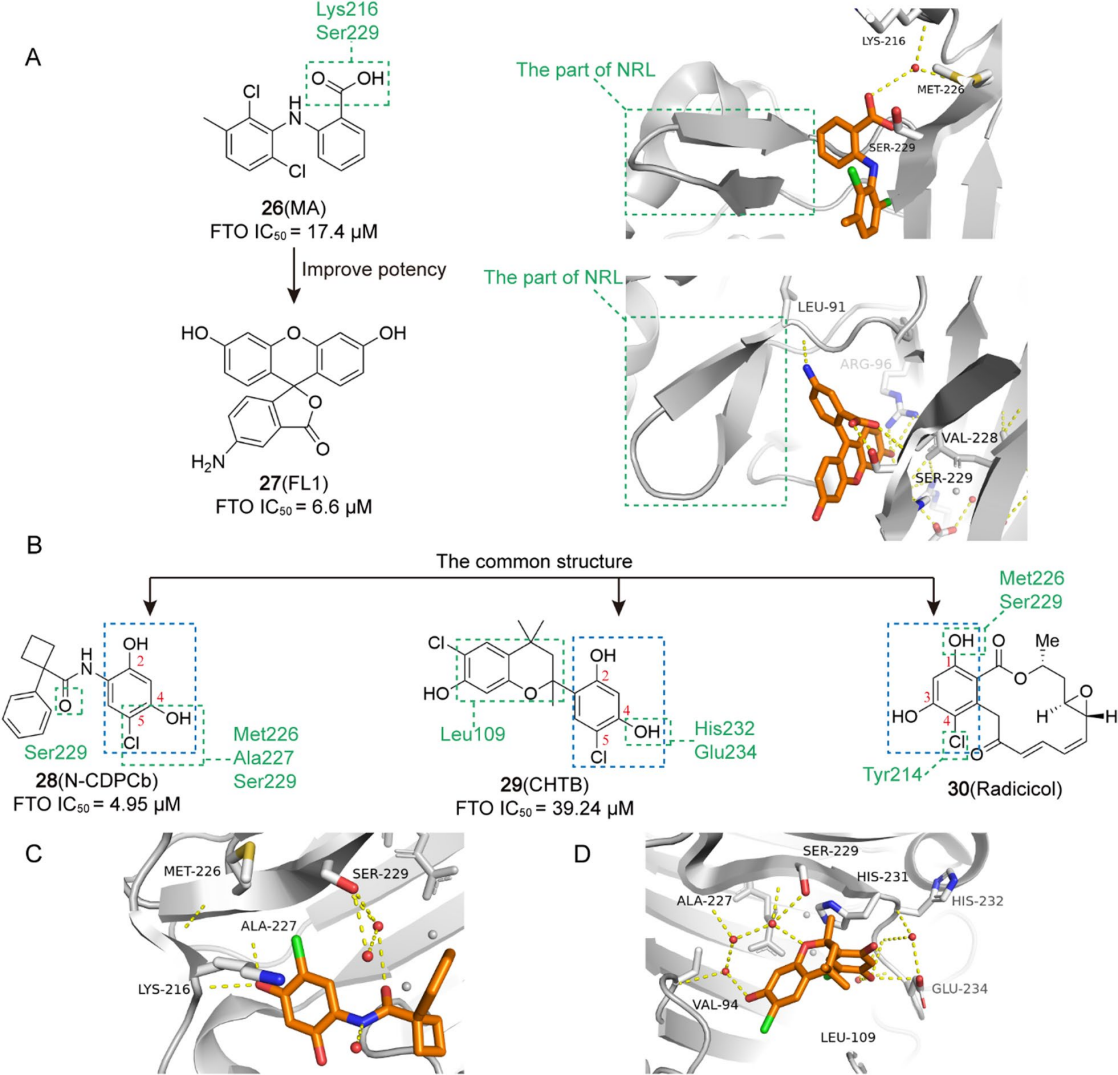
**A** Chemical structures of FTO inhibitors compound **26** and **27**, and the crystal structures of FTO in complex with compound **26** (PDB ID:4QKN) and **27** (PDB ID: 4ZS3), respectively. **B** Chemical structures of FTO inhibitors compound **28**–**30** containing the common structure 4-Cl-1, 3-diol group. **C** The crystal structures of FTO in complex with compound **28** (PDB ID: 5DAB). **D** The crystal structures of FTO in complex with compound **29** (PDB ID: 5F8P)

To improve the selectivity of FTO inhibitors, Huang's group [217] employed high-throughput fluorescence polarization (FP) experiments to examine compound selectivity differences against FTO and ALKBH5. They finally identified a specific FTO inhibitor compound **26** (Meclofenamic acid, MA, FTO IC_50_ = 17.4 μM) (Fig. 10A). Structurally, the crystal structure of compound **26** binding to FTO is shown as L-shape, has a similar acidic group as compound **21**. The carbonyl group can form hydrogen bonds with both Ser229 and Lys216, leading to the hypothesis that the carboxyl group is a crucial component for maintaining the inhibitory activity of compound **26**. In addition, hydrophobic interactions between carboxylic acid substituents with phenyl rings and side chains of neighboring residues also stabilize the binding structure of FTO to the compound **26**. Finally, the hairpin motif revealed by the FTO/MA complex as part of the FTO nucleotide recognition lid (NRL), which is missing in ALKBH5, results in the selectivity of compound **26** for FTO. In summary, compound **26** is neither a 2OG analogue nor a Fe^2+^ chelator but exerts highly selective FTO inhibitory activity in vitro by competing for FTO binding to ssDNA. In another study [218], the combination of compound **26**/gefitinib (GE) demonstrated a significant synergistic effect in GE-resistant NSCLC cells, overcoming GE resistance while inducing apoptosis. The mechanism may be related to the FTO inhibition by compound **26**, resulting in increased m^6^A levels and reduced mRNA levels of BC resistance protein (BCRP) and multidrug resistance protein-7 (MRP-7). Inspired by the FTO inhibitor compound **26**, Wang [219] developed a fluorescein derivative compound **27** (FL1, FTO IC_50_ = 6.6 μM) (Fig. 10A) in addition to the function of specific photoaffinity labeling to inhibit FTO activity. Similar to compound **26**, the selectivity of compound **27** from the hydrophobic NRL motif, hydrogen bonding targeting the carboxyl group, and hydrophobic effects targeting the phenyl structure together stabilized inhibitor binding to FTO. The crystal structure also shows that compound **27**/FTO has a large overlap with the binding site of compound **26**/FTO, suggesting that they may act on the same site. Due to its combined fluorescence labeling functionality and FTO inhibitory activity, compound **27** may serve as a potential tool molecule, facilitating research on the role of FTO in tumor-related signaling pathways.

Most of the previously mentioned inhibitors target the conserved 2OG related site of FTO. To further probe and improve the activity of FTO inhibitors, He [220] identified a novel inhibitor compound **28** (N-CDPCb, FTO IC_50_ = 4.95 μM) (Fig. 10B) that targets the non conserved binding site of FTO. Based on the crystal structure of FTO with mononucleotides, the investigators speculated that derivatives of benzene-1,3-diol could inhibit FTO activity as analogues of mononucleotides. Investigation of the structure–activity relationship revealed that introduction of both strong electron withdrawing groups and chlorine/bromine on the left-hand side phenyl ring impaired the inhibitory activity of the compounds. In the compound **28**/FTO complex, hydrogen bonds between the carbonyl oxygen and Ser229, Van Der Waals forces between the chloride and Met226, Ala227, and Ser229, and hydrophobic interactions formed by the cyclobutane ring and phenyl ring with the FTO fragment together mediate the specific binding of compound **28** to FTO. And the binding site is far from the traditional 2OG site and does not cause FTO conformational change when bound to FTO. Eventually, compound **28** was confirmed as a competitive inhibitor of FTO. Interestingly, although compound **28** is quite different from compound **26** structure, both of them presented L-shape spatially, with partial overlap. After discovering the new FTO binding site of compound **28**, Qiao [221] was inspired by it and continued to screen, and compound **29** (CHTB, FTO IC_50_ = 39.24 μM) (Fig. 10B) was identified as a new effective FTO inhibitor. Molecular docking showed that 4-OH can form hydrogen bonds with both His232 and Glu234, and 2-OH is not involved in the interaction with FTO. In addition to the strong hydrophobic contacts of the phenyl ring with His231 and Leu109, the chroman ring also interacts with Leu109. Both chlorine atoms in the compound also mediate Van Der Waals forces, collectively contributing to the stability of the CHTB/FTO complex. In addition, compound **29** also partially overlaps with compound **26**, similar to compound **28**. This suggests that there is a spatial structural similarity between the inhibitors we target close binding sites of FTO. In conclusion, compound **29** acts as a competitive inhibitor to reduce FTO enzyme activity in a dose-dependent manner. The discovery of compound **28** and compound **29** attracted the interest of Wang [222], through further SAR studies, who found 4-Cl-1,3-diol to be a consensus structure for such compounds. Here, based on a new binding site for FTO, researchers performed structure based virtual screening on compounds containing 4-Cl-1,3-diol groups and found that compound **30** (Radicicol) was a potent inhibitor of FTO (Fig. 10B).

In summary, compounds **28**–**30** act on the same site of FTO, and the structural similarities of 4-Cl-1,3-diol are their common features. The hydrogen bonds between hydroxyl groups and residues linked, the hydrophobic bonds between aromatic rings and residues linked, and the Van Der Waals forces between chlorine atoms and residues contributing collectively to their selectivity for FTO. For AML cells, FTO promotes the cell cycle, inhibits apoptosis, and enhances autophagic activity, thereby promoting the survival of leukemia cells. Therefore, inhibiting FTO through such small molecules may offer a promising approach in combating leukemia.

**Fig. 11:**
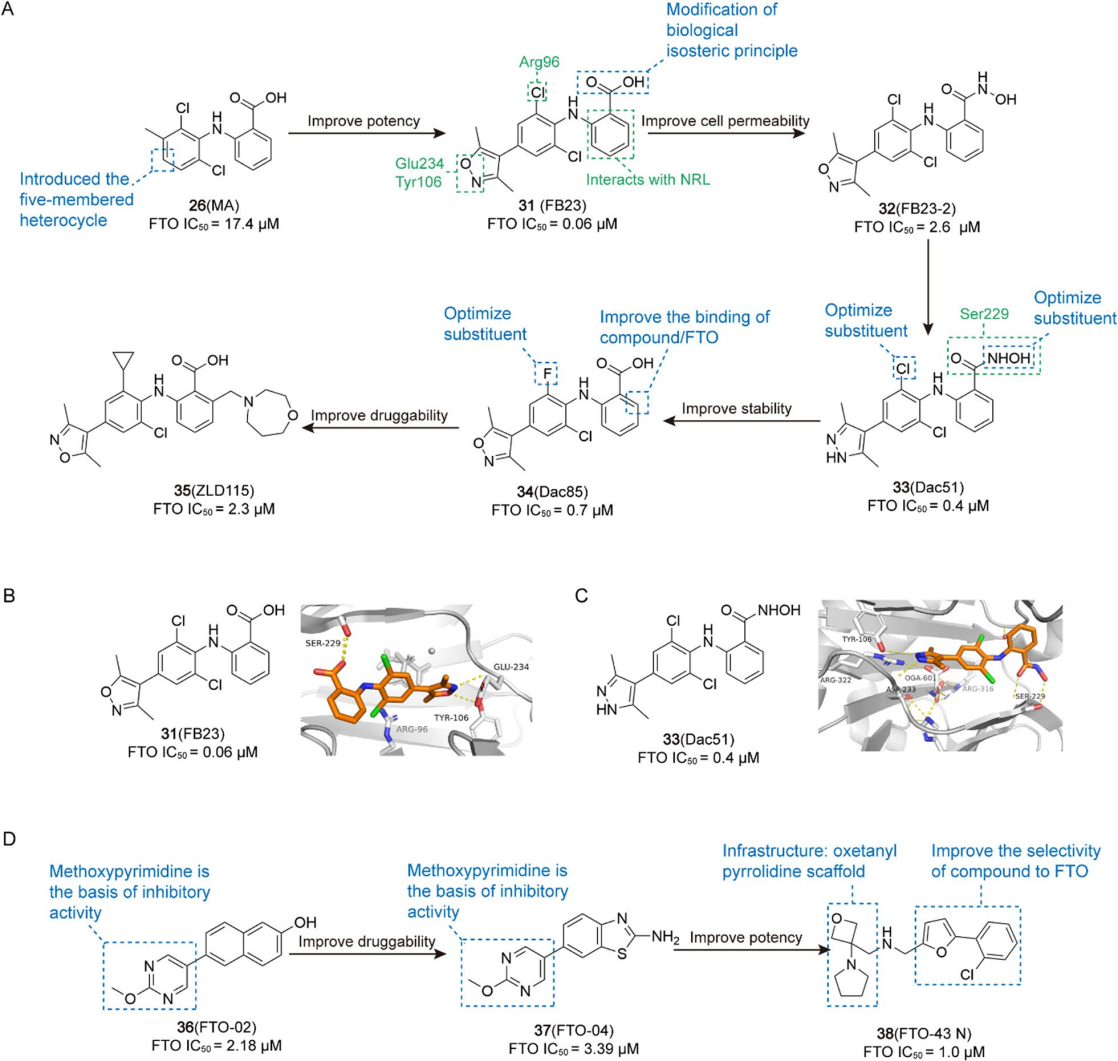
**A** Design and optimization process of FTO inhibitors compound **31**–**35**. **B** The crystal structure of FTO in complex with compound **31** (PDB ID: 6AKW). **C** The crystal structure of FTO in complex with compound **33** (PDB ID: 7CKK). **D** Design and optimization process of FTO inhibitors compound **36–38**

There are some FTO inhibitors, which may have various problems in terms of selectivity, inhibitory activity and ADME when they were first proposed. After constant iterations from researchers, their various properties are continuously converging towards clinical drugs. After developing compound **26** [217], a specific inhibitor of FTO, Huang's team further investigated SAR of FTO inhibitors. In 2019, the new specific and cell active FTO inhibitor compound **31** (FB23, FTO IC_50_ = 0.06 μM) and compound **32** (FB23-2, FTO IC_50_ = 2.6 μM) (Fig. 11A) were reported [15]. On the basis of retaining benzyl carboxylic acid, the inhibitory active component of compound **26**, the investigators introduced five membered heterocycles on dichlorobenzene. Crystallographic analysis found that the carboxyl bearing phenyl ring of compound **31** can interact with NRL to produce selectivity for FTO. Chlorine atom energy contacts bind the guanidinium group in Arg96. While the carboxyl group forms a hydrogen bond with the FTO residue side chain, in addition, the hydrogen bond also occurs between the N or O of the heterocycle and the amide backbone of Glu234. The extra hydrogen bonds generated on the heterocycles may be responsible for the improved inhibitory activity of compound **31**. The introduction of heterocycles helped compound **31** to occupy the pocket of FTO. Although compound **31** showed superior FTO inhibitory activity, its intracellular uptake was extremely low in NB4 and MONOMAC6 cells, which severely compromised the inhibition (NB4 IC_50_ = 44.8 μM, MONOMAC6 IC_50_ = 23.6 μM). In order to improve this situation, the researchers modified the structure on the basis of compound **31** to obtain compound **32**, a compound with slightly lower inhibitory activity but a greatly increased cell uptake rate. Taking advantage of the bioisosteric principle, the benzyl carboxyl group was engineered into a benzohydroxamic acid, and the intramolecular hydrogen bond between the amino hydrogen and the hydroxamic acid carbonyl may be responsible for its easier cellular entry. In terms of anti-tumor, compound **32** inhibited AML cell proliferation (IC_50_ = 1.6 ~ 16 μM), induce apoptosis and upregulate ASB2 and RARA, direct targets of FTO, to exert anti-tumor effects. The excellent properties of compounds **31** and **32** attracted interest from Liu, who further optimized them to develop more potent FTO inhibitor compound **33** (Dac51, FTO IC_50_ = 0.4 μM) [20] (Fig. 11A). Crystallographic analysis identified the presence of an additional hydrogen bond between the hydroxamic acid of compound **33** and Ser229 as the reason for the significant enhancement of compound **33**/FTO binding compared to compound **31**/FTO. By inhibiting FTO and increasing the methylation level of transcripts, compound **33** can inhibit the glycolytic capacity of tumor cells and exert antitumor proliferation activity. While the modification of hydroxamic acids increases the inhibitory activity and cell permeability of drugs, the instability of hydroxamic acids in metabolism similarly requires attention. To address this pitfall, Liu designed new FTO inhibitor compound **34** (Dac85, FTO IC_50_ = 0.7 μM) [21] (Fig. 11A) based on the SAR of tricyclic benzoic acids. Among the tricyclic benzoic acids, the introduction of five membered heterocycles generally showed higher inhibitory activity than the introduction of six membered rings, presumably the effect of the spatial structure of the FTO pocket. On the middle phenyl ring, neither substituents nor large sterically hindered substituents would affect the overall inhibitory activity of the compounds. Finally, the introduction of substituents on the phenyl ring of benzoic acid decreases the inhibitory activity. Taking the effects of cell permeability into consideration again, researchers finally designed compound **34.** Under the premise of similar inhibitory activity, compound **34** possesses excellent cell permeability, which can inhibit AML cells and exert an anti-leukemia effect. The rigid chemical structure of compound **34** may affect its drug resistance, causing hindrance to subsequent drug development. To this end, Xiao [223] optimized tricyclic benzoic acid to tetracyclic benzoic acid and designed a new FTO inhibitor compound **35** (ZLD115, FTO IC_50_ = 2.3 μM) (Fig. 11A). Introduction of a flexible base side chain at the solvent accessible position 6 of benzoic acid improved binding of the compound to the FTO protein. Although inhibitory activity was strongest when a five membered nitrogen heterocycle was introduced, the lipophilicity of the compound did not meet the criteria at this time, so the benzylic 1,4-oxazepan group was finally chosen as the introduction group. Furthermore, based on the combined considerations of inhibitory activity and ADME, one Cl on the phenyl group was replaced with a cyclopropyl group. The flexible side chains help the compound to better occupy the FTO pocket, inhibiting its action by competing for the substrate of FTO. In terms of drug efficacy, compound **35**, which is metabolically stable in vivo with high permeability and no obvious cytotoxicity, had a good inhibitory effect on the proliferation of AML cells by targeting FTO. In terms of mechanism, compound **35** can upregulate RARA and downregulate MYC levels in MOLM13 cells, inhibiting the oncogenic FTO signaling pathway in AML cells, thereby demonstrating anti-leukemic activity. In animal experiments, female BALB/c nude mice transplanted with human myeloid monocytic leukemia cell MV-4-11 had elevated m^6^A levels, generally smaller tumor sizes than controls, and demonstrated good biosafety after compound **35** administration. This means that compound **35** has taken a solid step towards qualifying anti-tumor drugs.

By analyzing the binding site of compound **26** with FTO, Sarah Huff [224] et al. searched for potential compounds to assist in the development of FTO inhibitors and finally screened a competitive inhibitor of FTO, compound **36** ((FTO-02, FTO IC_50_ = 2.18 μM) and compound **37** (FTO-04, FTO IC_50_ = 3.39 μM) (Fig. 11D). By screening the rationally designed compounds for inhibitory activity, it was found that compounds containing 2-methoxypyrimidine or 6-methoxynaphthalene exhibited superior inhibitory activity. In cellular assays, compound **37** exerts antitumor effects by increasing m^6^A levels in cells and interfering with GSC renewal, leading to new clues for the treatment of GBM. After developing compound **37**, Huff [13] was further optimized on the basis of improved selectivity and inhibitory activity to design the oxetanyl FTO inhibitor compound **38** (FTO-43 N, FTO IC_50_ = 1.0 μM) (Fig. 11D). Through structural screening, the oxetanyl pyrrolidine scaffold was finally identified as the base scaffold on which the optimization was carried out. Found that connecting the side chain between the furan and substituted benzene ring generally exhibits higher FTO selectivity compared to connecting indole. Based on this, further optimization can be performed with regard to the position and quantity of substituents on the benzene ring. Considering the selectivity, inhibitory activity and lipophilicity of the compounds together, it was found that the compounds performed best when the phenyl ring had only one Cl, thus confirming that compound **38** was the best choice. In subsequent studies of anti-tumor activity, compound **38** showed promising inhibitory activity against AML, GBM, and gastric cancer. In subsequent studies of anti-tumor activity, compound **38** effectively inhibited the growth of Gastric cancer AGS, KATOIII, and SNU-16 cell lines (AGS EC_50_ = 20.3 μM, KATOIII EC_50_ = 35.9 μM, SNU-16 EC_50_ = 17.7 μM), while exhibiting no growth toxicity to normal CCD841-CoN colon cells. This is associated with the downregulation of the Wnt and PI3K-Akt signaling pathways in gastric cancer cells induced by treatment with compound **38**.

**Fig. 12:**
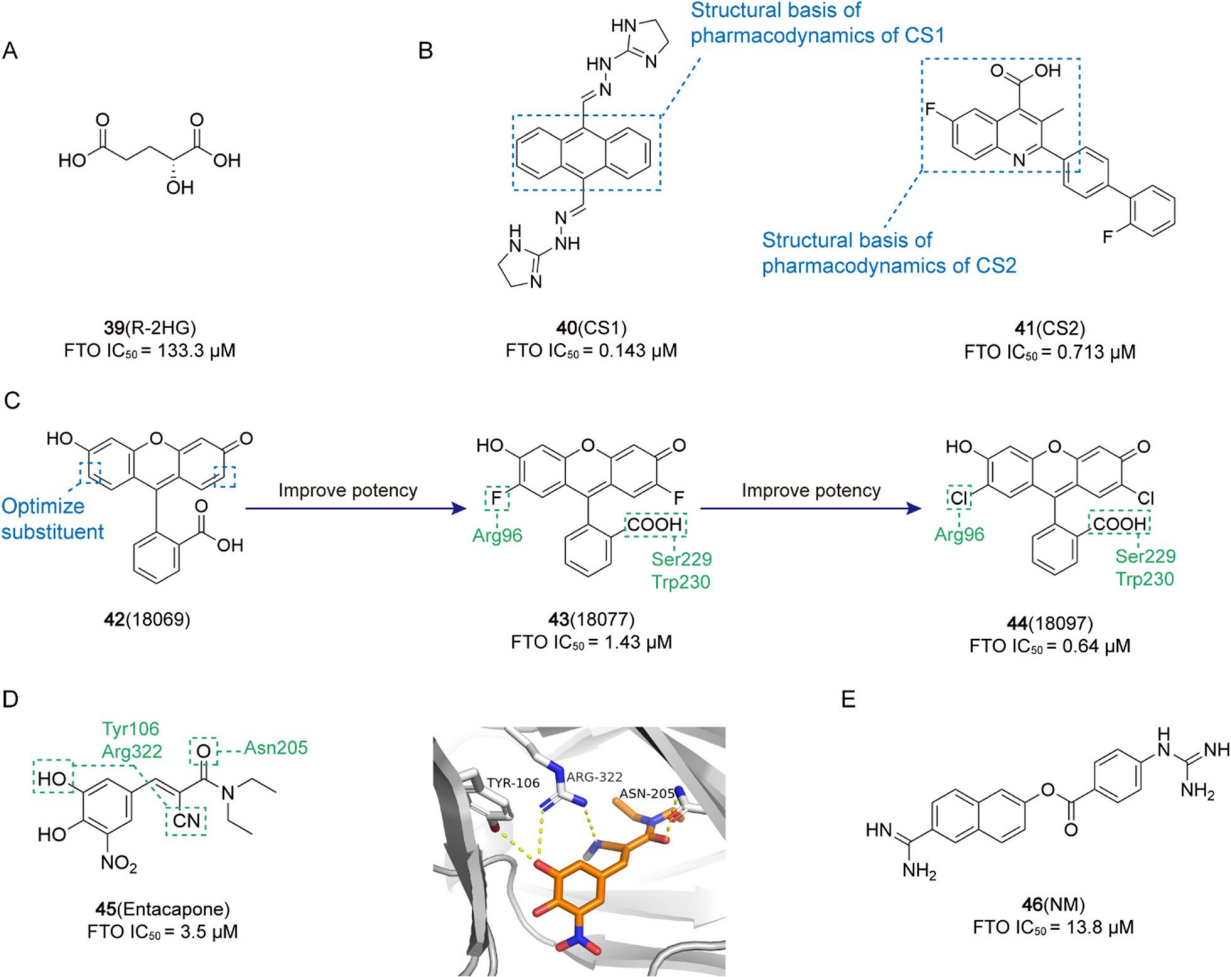
**A** Chemical structure of FTO inhibitor compound **39** based on the design of an intermediate metabolite. **B** Chemical structures of effective FTO inhibitors compound **40** and **41** based on virtual screening. **C** Design and optimization process of FTO inhibitors compound **42**–**44**. **D** Chemical structure of FTO inhibitor compound **45** and the crystal structure of FTO in complex with compound **45** (PDB ID: 6AK4). **E** Chemical structure of FTO inhibitor compound **46**

Finding inhibitors of FTO in addition to structure based drug design, getting started with the human active metabolite perspective is a promising approach. An interesting phenomenon that has caught the attention of researchers is that patients with isocitrate dehydrogenase (IDH)—mutated GBM and AML have better survival than those without IDH lesions [225]. Further investigation revealed that this interesting phenomenon may be related to R-2HG, a metabolite of IDH, compound **39** (R-2HG, FTO IC_50_ = 133.3 μM) (Fig. 12A) can affect the FTO/m^6^A/MYC/CEBPA pathway and inhibit tumor proliferation and survival by repressing the expression of FTO. Mechanistic studies revealed that compound **39** could inhibit FTO activity, increase the methylation levels of c-Myc and CEBPA mRNA, and reduce the stability of their transcripts, thereby suppressing related signals and inhibiting tumor progression. This suggests that our structure optimization based on compound **39** may be a promising direction for FTO inhibitor research. Although a number of inhibitors have been reported previously for FTO, some problems exist such as weak inhibitory activity, weak cellular activity, and unclear antitumor efficacy. To this end, Su [226] identified two highly potent FTO inhibitors, compound **40** (CS1, FTO IC_50_ = 0.143 μM) and compound **41** (CS2, FTO IC_50_ = 0.713 μM) (Fig. 12B). Both of them exhibit excellent antileukemic activity in vitro and in vivo and are involved in regulating the expression of immune checkpoint genes. In compound **40**, the planar tricyclic structure is the basis for maintaining its drug efficacy, and the N on the heterocycle can interact with FTO residues. In compound **41**, the carboxylic acid hydroxyl group and the aromatic ring structure of quinoline together with the N on the ring participate in the stabilization of the compound **41**/FTO complex, allowing both compound **40** and **41** to bind tightly to the catalytic pocket of the FTO protein. Mechanistic studies revealed that FTO inhibition mediated by compound **40** and **41** can sensitize leukaemia cells to T cell cytotoxicity and overcome hypomethylation induced immune evasion.

Xie [14] reported two selective FTO inhibitors with cellular activity, compound **43** (18,077, FTO IC_50_ = 1.43 μM) and compound **44** (18,097, FTO IC_50_ = 0.64 μM) (Fig. 12C). Based on virtual screening and compound design, researchers first designed the basic framework compound **42** (18,069), and subsequent studies found that halogen substitution can improve the selectivity and inhibitory activity of compounds. Molecular modeling found that compound **43** and **44** can occupy the substrate binding site of FTO. First, they can form hydrogen bonds with Arg96, Ser299, and Trp230 residues. Second, the halogen atom is then connected to Arg96. Finally, the hydrophobic interaction of the phenyl ring with NRL mediates the selectivity in targeting FTO. In anti-tumor studies, compound **44** not only inhibited BC cell MDA-MB-231 proliferation, metastasis and EMT through metabolic reprogramming, but also inhibited apoptosis and improved chemosensitivity, suggesting its promise as a potential therapeutic agent for BC.

Recently, some already marketed drugs have also been found to inhibit FTO activity. Compound **45** (Entacapone, FTO IC_50_ = 3.5 μM) (Fig. 12D) is a COMT inhibitor for the treatment of Parkinson's. Recently, its FTO inhibitory activity was reported by Peng [227]. Structurally, the hydroxyl group is an essential group to maintain inhibitory activity, and either the removal of the hydroxyl group or the use of methoxy substitution decreases inhibitory activity. Since the nitro group lacks interaction with protein structure, replacing the nitro group might be a way to further improve the activity of the compound. Furthermore, engineering the flexible diethyl tail into an alicyclic ring is also a good way to improve the inhibitory activity. In addition to compound **45**, compound **46** (Nafamostat mesilate, NM, FTO IC_50_ = 13.8 μM) (Fig. 12E) as a serine protease inhibitor also possesses FTO inhibitory activity [228]. Consistent with previous studies [216], Glu234 is an important residue for stabilizing the compound **46**/FTO complex, besides which the aromatic ring of the compound and the amidines at both ends are involved in hydrophobic interactions and hydrogen bond formation. Activity assays showed that compound **46** is a dose-dependent FTO inhibitor. Previous studies have shown that compound **46** inhibits both the NF-κB pathway and colorectal cancer growth and metastasis. It can also be combined with oxaliplatin in the treatment of pancreatic cancer [229, 230]. Now, the newly discovered targets of compound **46** may extend it to new tumor types.

Due to their early discovery and abundance drug targets, FTO inhibitors represent the most mature and diverse category of research. In the future, these small molecules should continue to be explored for their anti-cancer potential in advancing towards clinical trials.

### Targeting readers

#### YTHDFs inhibitors

**Fig. 13:**
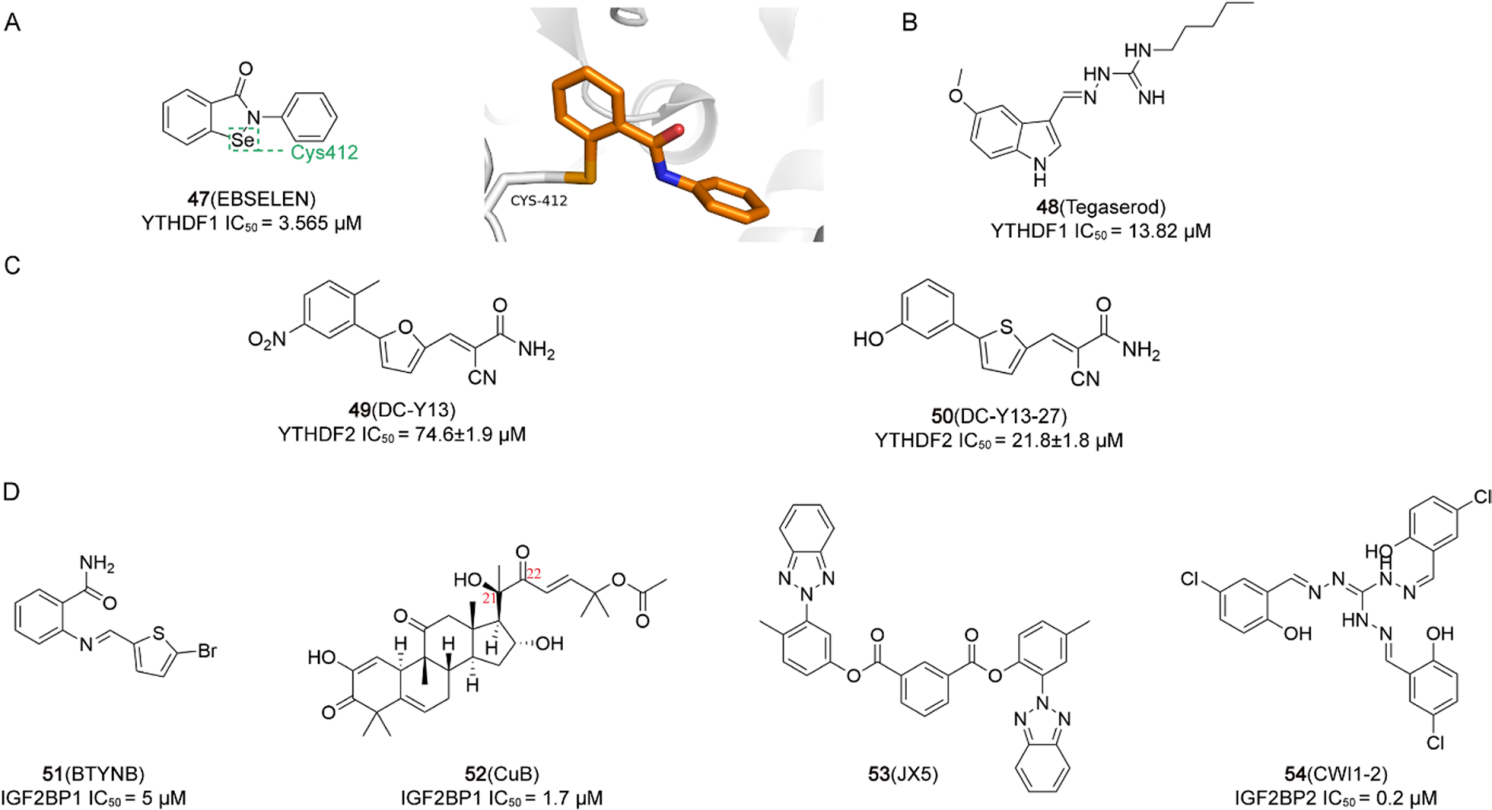
**A** Chemical structure of YTHDF1 inhibitor compound **47** and the crystal structure of YTHDF1 YTH domain in complex with compound **47** (PDB ID: 7PCU). **B** Chemical structure of YTHDF1 inhibitor compound **48. C** Chemical structure of YTHDF2 inhibitors compound **49** and **50**. **D** Chemical structure of IGF2BPs inhibitors compound **51**–**54**

As m^6^A readers, the YTH domain can recognize m^6^A modified sites in a methylation dependent manner [58, 231, 232]. Previously, the crystal structure of the YTHDFs/RNA complex was revealed [233, 234], which revealed the structural basis for the function of the YTH domain and provided the necessary conditions for the design of YTHDFs inhibitors. In 2022, Micaelli [17] designed YTHDF1 inhibitor compound **47** (Ebselen, YTHDF1 IC_50_ = 3.565 μM, EC_50_ = 1.63 μM) (Fig. 13A) based on high-throughput screening. Mechanically, compound **47** can bind to the YTH domain of YTHDF1, hinder the binding of YTHDF1 to RNA and inhibit the recognition of m^6^A-modified RNA. On the crystal structure, the Se atom of compound **47** can covalently bind with Cys412, while benzamide can interact with the domain constituted by the residue. Based on the nature of the environment, compound **47** binds to the m^6^A pocket of the YTHDF1 protein in a covalent or non-covalent form, exerting YTHDF1 inhibitory activity. Furthermore, given that the effective concentrations of compound **47** for YTHDF1 and YTHDF2 are quite close (EC_50_ = 1.63 μM and 1.66 μM), which is not selective for either protein. Finally, cell experiments revealed that compound **47** exhibited time-dependent inhibitory effects on PCa cells (24h, IC_50_ = 58.77 μM; 48h, IC_50_ = 49.58 μM; 72h, IC_50_ = 26.83 μM). As a treatment for irritable bowel syndrome with constipation, compound **48** (Tegaserod, YTHDF1 IC_50_ = 13.82 μM) (Fig. 13B) was recently reported as a novel potential YTHDF1 inhibitor, which prevents YTHDF1 from binding m^6^A modified mRNA and YTHDF1 dependent protein translation. Cell based assays revealed that compound **48** was able to target YTHDF1 in a dose-dependent manner and reduce THP-1 activity in AML cells in vitro (IC_50_ = 3.58 μM). In addition, for CD34 + cells, compound **48** also reduces the burden of AML by blocking the G1 phase to inhibit cell proliferation and inducing apoptosis. In 2023, Wang [235] performed fluorescence polarization based high-throughput screening on its internal compound library and discovered a YTHDF2 inhibitor compound **49** (DC-Y13, YTHDF2 IC_50_ = 74.6 ± 1.9 μM) (Fig. 13C). After further structural optimization, they obtained a more potent inhibitor, compound **50** (DC-Y13-27, YTHDF2 IC_50_ = 21.8 ± 1.8 μM, YTHDF1 IC_50_ = 165.2 ± 7.7 μM, K_D_ = 37.9 ± 4.3 μM) (Fig. 13C). Since the inhibitory activity of compound **50** against YTHDF2 is much greater than that against YTHDF1 and does not inhibit the expression of the YTHDF1 target LRPAP1, it can be considered that compound **50** possesses selectivity for YTHDF2. Found that compound **50** alone don’t have anti-tumor efficacy in MC38 or B16 mouse models, possibly because of its weak YTHDF2 inhibitory activity. But compound **50** plays an important role in combination therapy by significantly enhancing the tumor suppressive effect of ionizing radiation (IR), and that compound **50** also exhibited a function of enhancing anti-PD-L1 efficacy in MC38 models. Mechanistically, compound **50** could reverse IR induced MDSC increase in immunosuppressive cells and enhance body adaptive immunity to assist IR in combination therapy by suppressing YTHDF2 expression. In the future, further optimization of the inhibitory activity of compound **50** may bring more hope for anti-tumor therapy.

Due to the limited research duration, small molecule inhibitors targeting YTHDFs reported to date are facing challenges in terms of inhibitory activity, cellular activity, and selectivity. However, they also hold significant potential for optimization, making it an area in need of further exploration with promising prospects.

#### IGF2BPs inhibitors

By mediating multiple signaling pathways, IGF2BP plays an oncogenic role in a variety of cancers, and inhibition of IGF2BP is considered an effective antitumor modality. In 2017, Mahapatra analyzed a large number of compounds based on fluorescence anisotropy and reported that the small molecule compound **51** (BTYNB, IGF2BP1 IC_50_ = 5.0 μM) (Fig. 13D). It is a potent and selective inhibitor against the interaction between IGF2BP1 protein and c-Myc mRNA [236, 237]. By downregulating multiple mRNA transcripts regulated by IGF2BP1, compound **51** can destabilize oncogenic c-Myc mRNA and inhibit ovarian cancer and melanoma cell proliferation. Among the natural products, the tetracyclic triterpenoid cucurbitacin B (compound **52**, CuB, IGF2BP1 IC_50_ = 1.7 μM) [238] (Fig. 13D) was found to target IGF2BP1 to exert inhibitory effects. It was found that compound **52** could covalently bind to the thiol of cysteine of Cys253 in IGF2BP1 proteins, and the αβ-unsaturated carbonyl group on the compound structure participated in this role. Whereas, the 21-hydroxyl and 22-keto groups also form hydrogen bonds with Gln341 and Ser254 residues, which makes the well matched binding site between compound **52** and the surrounding Cys253 in stereo configuration. In conclusion, compound **52** can block c-Myc mRNA recognition by IGF2BP1 through allosteric effects, inducing apoptosis, restoring the immune response, and exerting antihepatoma effects. IGF2BP2 can directly bind to NOTCH1 and stabilize NOTCH1 mRNA expression in an m^6^A dependent manner, and the widespread expression of NOTCH1 may be closely linked to the presence of T-cell acute lymphoblastic leukemia (T-ALL). To combat T-ALL, Feng developed an IGF2BP2 inhibitor compound **53** (JX5) [239] (Fig. 13D). Structurally, the aromatic and benzonitrogen heterocycles on compound **53** can interact with residues to mediate the tight binding of the compounds to the IGF2BP2 protein and hinder the binding of IGF2BP2 to NOTCH1, thereby exerting antitumor effects. In a cellular assay in Jurkat cells, compound **53** did not affect IGF2BP2 mRNA levels but significantly reduced NOTCH1 mRNA expression and triggered apoptosis similar to the effects of knocking down IGF2BP2. It should be noted that the therapeutic effect of compound **53** itself is not strong and has some cytotoxicity, therefore, further optimization of the selectivity versus safety of compound **53** is also needed. Weng [111] developed the IGF2BP2 inhibitor compound **54** (CWI1-2, IGF2BP2 IC_50_ = 0.2 μM) (Fig. 13D) with antileukemic activity through virtual screening. Structurally, the presence of a hydrophobic binding interaction of the substituted phenyl group with the KH4 domain of RNA brings compound **54** close to the RNA binding pocket of IGF2BP2, implying that the inhibitory activity of compound **54** on IGF2BP2 is competitive. Follow up studies showed that compound **54** exhibited excellent antileukemic effects in vitro and in vivo by targeting IGF2BP2.

Due to the recent discovery of many readers, small molecules targeting readers are still in their early stages of development. However, considering the diverse functions of readers, these related small molecules are expected to offer more diverse possibilities for cancer treatment.

## Conclusion and future prospects

As a crucial element of epigenetics, m^6^A modification can regulate the methylation of RNA at the post-transcriptional level, thereby influencing its expression and playing an important role in the normal progress of biological activities. Studies have found that m^6^A modification is dysregulated during the initiation and progression of multiple cancers. Recently, on the basis of elucidating the functions of m^6^A regulators, the relationship between m^6^A modification and cancer has been continuously explored, aiming to gain a deeper understanding of the significance of m^6^A modification in tumor proliferation, metastasis, drug resistance, immunotherapy, and prognosis. Moreover, due to the high specificity of small molecules targeting m^6^A modifications, their adverse effects on normal cells are minimal. These molecules offer an alternative approach to cancer treatment and provide a new avenue for addressing drug-resistant tumors that do not respond to traditional therapies. It can also be used in combination with other anti-tumor drugs to enhance efficacy, overcome resistance, and reduce adverse reactions. Research and development of small molecules targeting m^6^A are gradually emerging as promising antitumor strategies.

Currently, METTL3/14 and FTO have emerged as the most extensively reported m^6^A regulators during tumor development, with a majority of small molecule interventions targeting these two targets. From a small molecule discovery perspective, small molecules are primarily obtained through high-throughput screening of compound libraries, natural products, drug repurposing and structure optimization based on structure–activity relationships. From a mode of action perspective, a large proportion of small molecules are classified as competitive inhibitors that primarily hinder their activity mainly by inhibiting the binding of regulators and substrates. For example, in METTL3 inhibitors, compound **2** [196] is an adenosine analogue of SAM, whereas compound **6** (UZH1a) [12] occupies the SAM binding pocket. Among the FTO inhibitors, the substrate binding site of FTO, demethylation cofactor 2OG and Fe^2+^ are also all top targets. From the perspective of structure–activity relationship, these small molecules all snugly fit into the m^6^A modulator in the crystal structure and mainly occupy the cavity through hydrogen bonding, hydrophobic bonds, and Van Der Waals forces effectively. For example, FTO inhibitors with either a 4-Cl-1,3-diol structure or a tricyclic benzoic acid structure all exhibit good inhibitory activity. Based on these findings, we can be guided to better develop highly effective small molecules targeting m^6^A regulators. In addition, there exist a limited number of compounds targeting other m^6^A regulators, however, their inhibitory activities are relatively weak and necessitate further investigation.

Although many small molecules reported so far have promising inhibitory activities, there are also some issues that need to be addressed urgently. First, the intracellular activity of some small molecules still requires enhancement due to the selectivity of cell uptake. For example, the cellular uptake of compound **31** (FB23) by acute promyelocytic leukemia cells (NB4) and human acute monocytic leukemia cells (MONOMAC6) is limited, resulting in a low intracellular concentration that makes compound **31** only exhibit highly potent inhibitory activity extracellularly [15]. However, it only exhibited poor proliferation inhibition effect on NB4 (IC_50_ = 44.8 μM) and MONOMAC6 (IC_50_ = 23.6 μM). Reassuringly, subsequent introductions of small molecules such as compound **32** (FB23-2), compound **33** (Dac51) and compound **34** (Dac85) based on this optimization significantly improved cellular uptake. Second, most studies have been limited to examining the inhibitory activity of small molecules, however, their ADME properties, such as lipophilicity and solubility, remain to be optimized. Drugs entering clinical trials are also comparatively lacking, with only STC-15 [197], a derivative of the METTL3 inhibitor STM2457, currently in phase I clinical trials while the rest are still in preclinical studies. In addition, because of the wide variety of subtyping stages and different characteristics at different stages of cancer, the specific applicability of small molecules needs to be further clarified and subdivided. Although small molecules such as compound **21** (Rhein), compound **22** (Clausine E), compound **23** (Diacerein) and compound **29** (CHTB) exhibit FTO inhibitory activity, their therapeutic effects on specific cancers have not been reported. Some small molecules with excellent inhibitory activity and cellular activity, such as compound **35** (ZLD115), also lack discussion of effects for specific cancer subtypes. Finally, the research strategies for targeting m^6^A small molecules are primarily limited to screening of natural products, Computer-Aided Drug Design (CADD) and drug repurposing. However, there is a scarcity of designs based on structure–activity relationships and inadequate application of new methods such as activity-based protein profiling (ABPP), thermal proteome profiling (TPP), PROTAC and molecular glues. Additionally, there is a lack of integration with prominent areas in antitumour research such as ferroptosis, immunecheckpoint inhibitor (ICI) and synthetic lethality.

**Fig. 14:**
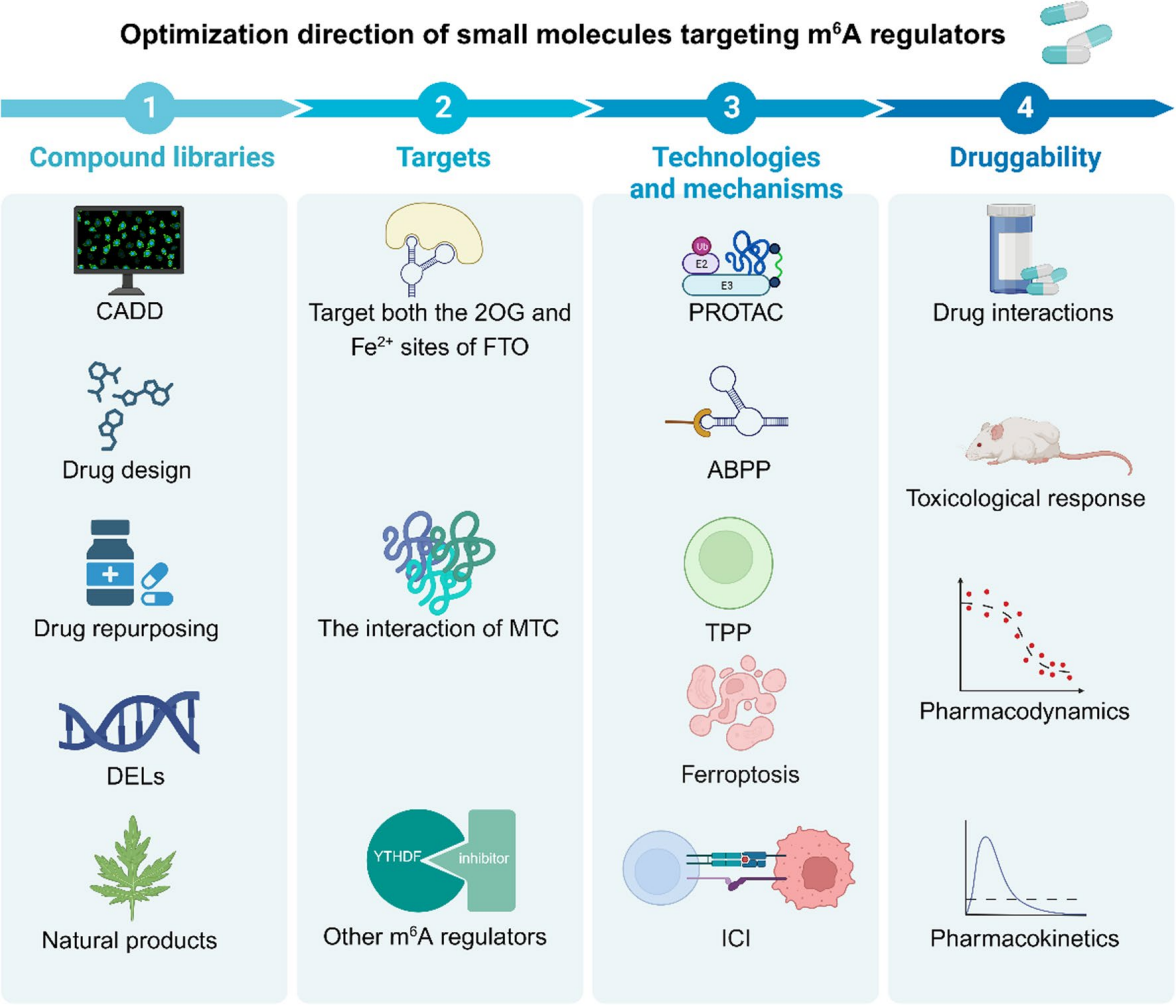
The development direction of small molecules targeting m^6^A regulators. Build compound libraries based on CADD, drug design, drug repurposing, DNA-Encoded Libraries (DELs) and natural products. Compounds can then be screened for potential new targets (eg. multiple targets targeting FTO simultaneously, targeting interactions between MTCs, targeting other less studied m^6^A regulators), and then applying PROTAC, ABPP, TPP, ferroptosis, ICI and other technologies or mechanisms to optimize compounds. Finally, the druggability of compounds should be improved as much as possible

Combined with the issues mentioned earlier, future directions of small molecules targeting m^6^A can be envisioned. Firstly, it is imperative to establish high-quality compound libraries sourced from diverse origins. Utilizing Artificial Intelligence (AI) and Machine Learning (ML) technologies, such as the DEREPLICATOR^+^ and DP4-AI tools based on mass spectrometry and NMR, allows for the rapid identification and elucidation of compound structures from complex samples. Tools like NPClassifier can deeply categorize compounds based on their biosynthetic pathways and chemical properties, further refining the scope of candidate drugs [240–242]. Secondly, building upon the improved understanding of the mechanisms governing m^6^A modification, we can pursue the development of more small molecules. These molecules should either target other regulators of m^6^A or elicit synergistic effects by simultaneously addressing multiple targets. For example, designing small molecules that can act on both the FTO Fe^2+^ site and 2OG binding site, designing small molecules that act on the interaction between MTC, stitching the existing small molecules into twin drugs. Moreover, the application of emerging technologies offers avenues for refining the selectivity and efficacy of these small molecules. Employing ABPP technology to pinpoint more efficient enzyme targets, utilizing TPP to identify targets with robust cellular activity, harnessing PROTAC [243, 244] or molecular glues to directly degrade m^6^A regulator proteins, alongside exploring small molecules that interfere with MTC complex interactions, thus strategically influencing the dynamic equilibrium of m^6^A modification. Moreover, the application of AI and ML technologies in biological activity and target identification, through tools like DEEP Picker for NMR spectroscopy analysis, accelerates the discovery of potential drugs, allowing for the early prediction of the biological effects of compounds, providing key support for drug development [240]. Furthermore, a deeper investigation into the relationship between m^6^A modifications and tumor progression opens new avenues for combination therapy strategies. Specifically, integrating m^6^A inhibitors with first-line treatments and ICI not only modulates the TME to enhance therapeutic response but also effectively addresses treatment resistance across various cancer types. With continued research, such innovative treatment approaches are anticipated to be applied in practice, potentially leading to breakthroughs in cancer therapy. Finally, the druggability of small molecules can be continuously enhanced, and the properties of compounds can be comprehensively investigated from various perspectives including pharmacodynamics, pharmacokinetics, and toxicology (Fig. 14).

In summary, extensive research has been conducted on m^6^A modification in the context of cancer [245–247], and there is growing interest in utilizing small molecules targeting m^6^A as potential antitumor drugs. This article provides a comprehensive overview of recent advancements in relevant small molecules and anticipates the emergence of more efficient and selective compounds in the future, thereby offering new prospects for tumor therapy.

## Data Availability

The material supporting the conclusions presented in this review has been included within the article.

## Abbreviations

m^6^A: N^6^-methyladenosine
METTL3: Methyltransferase-like 3
METTL14: Methyltransferase-like 14
METTL4: Methyltransferase-like 4
METTL5: Methyltransferase-like 5
METTL16: Methyltransferase-like 16
WTAP: Wilms tumor 1-associated protein
RBM15/RBM15B: RNA-binding motif protein 15
VIRMA: Vir-like m^6^A methyltransferase associated
CBLL1: Cbl proto-oncogene-like 1
ZC3H13: Zinc fnger CCCH-type containing 13
ZCCHC4: Zinc fnger CCCH-type containing 4
PCIF1: Phosphorylated CTD interacting factor 1
FTO: Fat mass- and obesity- associated protein
ALKBH5: ALKB homolog 5
YTHDF: YTH domain family protein
YTHDC: YTH domain-containing protein
HNRNPA2B1: Heterogeneous nuclear ribonucleoproteins A2/B1
HNRNPC: Heterogeneous nuclear ribonucleoproteins C
HNRNPG: Heterogeneous nuclear ribonucleoproteins G
eIF3: Eukaryotic translation initiation factor 3 subunit A
IGF2BP: Insulin-like growth factor 2 mRNA-binding protein
FMRP: Fragile X mental retardation protein
PRRC2A: Proline rich coiled-coil 2A
EMT: Epithelial-to-mesenchymal transition
BC: Breast cancer
AML: Acute myeloid leukemia
GBM: Glioblastoma
HCC: Hepatocellular carcinoma
CRC: Colorectal cancer
LC: Lung cancer
SCLC: Small cell lung cancer
NSCLC: Non-small cell lung cancer
PCa: Prostate cancer
carRNAs: Chromosome-associated regulatory RNAs
MYC: Myelocytomatosis viral oncogene
Bcl-2: B-cell lymphoma-2
MDSC: Myeloid-derived suppressor cell
SRSF: Serine/arginine-rich splicing factor
NOTCH: Neurogenic locus notch homolog protein
HIF: Hypoxia-inducible factors
3'-UTRs: 3 '- Untranslated regions
ASB2: Ankyrin repeat and SOCS box containing 2
RARA: Retinoic acid receptor alpha
ADME: Absorption, distribution, metabolism and extraction
SAR: Structure–activity relationships
SAM: S-adenosylmethionine
MTC: Methyltransferase complex
ECJs: Exon junction complexes
TFs: Transcription factors
TRMT112: TRNA methyltransferase activator subunit 11–2
ssRNAs: Single stranded RNAs
ncRNAs: Non coding RNAs
snRNA: Small nuclear RNA
hm6A: Hydroxymethyladenosine
f6A: Formyladenosine
m6Am: N6-2'-O-dimethyladenosine
LSCs: Leukemia stem cells
pri-miRNAs: Primary microRNAs
RGG: ArgGly-Gly
RNAPII: RNA polymerase II
RRMS: RNA recognition motifs
TME: The tumor microenvironment
AURKA: Aurora kinase A
BCSC: Breast cancer stem cell
CDKN1A: Cyclin-dependent kinase inhibitor 1a
mdm2: Mouse double minute 2
GSCs: Glioblastoma Stem Cells
DLL3: Delta like ligand 3
HES1: Hairy and enhancer of split 1
PDGF: Platelet derived growth factor
OPTN: Optineurin gene
SOCS2: Suppressor of cytokine signaling 2
HBXIP: Hepatitis B x-interacting protein
LNC942: LINC00942
CXCR4: C-X-C motif chemokine receptor 4
RMRP: RNA component of mitochondrial RNA processing endoribonuclease
YBX1: Y-box binding protein 1
TGFBR1: Transforming growth factor beta receptor 1
XIST: X-inactive specific transcript
ETS1: ETS proto oncogene 1
BCAA: Branched chain amino acid
BCAT1: Branched chain amino acid transaminase 1
eIF4E2: Eukaryotic translation initiation factor 4E family member 2
eIF4E: Eukaryotic translation initiation factor 4E
GATA3: GATA binding protein 3
NANOG: NANOG homeobox gene
EIF4EBP1: Eukaryotic translation initiation factor 4e binding protein1
TCF15: Transcription factor 15
LSCs/LICs: Stem/initiating cells
ITPA: Inosine triphosphatase
LYPD1: Ly6/PLAUR domain-containing protein 1
NPM1: Nucleophosmin 1
ERK: Extracellular signal regulated kinase
TP53INP2: Tumour protein p53 inducible nuclear protein 2
pri-miR-10a: Primary miRNA-10a
FOXO3a: Forkhead box O3
HK2: Hexokinase 2
EZH2: Enhancer of zeste homolog 2
CREB1: Cyclic AMP response binding protein 1
STEAP3: STEAP3 metalloreductase
GSK3β: Glycogen synthase kinase 3β
SLC1A5: Solute carrier family 1 member 5
RCC2: Regulator of chromosome condensation 2
MSI1: Musashi-1
LXRA: Liver X receptor A
HIVEP2: Hivep zinc finger 2
IL11: Interleukin 11
SERPINE2: Serpin family e member 2
LHPP: Phospholysine phosphohistidine inorganic pyrophosphate phosphatase
NKX3-1: NK3 homeobox 1
nYACs: Nuclear YTHDC1-m6A condensates
MALAT1: Metastasis associated lung adenocarcinoma transcript 1
F. nucleatum: Fusobacterium nucleatum
NFIB: Nuclear factor I B
USP4: Ubiquitin specific peptidase 4
ELAVL1: ELAV like RNA binding protein 1
ARHGDIA: Rho GDP dissociation inhibitor alpha
CDK19: Cyclin-dependent kinase 19
Pol II Ser2: RNA polymerase II serine 2
RUNX2: Runt-related transcription factor 2
TNBC: Triple-negative breast cancer
TGF-β1: Transforming growth factor-beta 1
ARHGEF2: Rho/RAC guanine nucleotide exchange factor 2
FLT4: Fms related tyrosine kinase 4
ABC: ATP binding cassette
MDR: Multidrug resistance
ESRRG: Estrogen-related receptor gamma gene
ERRγ: Estrogen-related receptor gamma
ABCB1: ATP-binding cassette subfamily B member 1
CPT1B: Carnitine palmitoyltransferase 1B
ITGA4: Integrin alpha 4
MSaCs: Mesenchymal stem cells
HER2: Human epidermal growth factor receptor 2
COX6B2: Cytochrome C oxidase subunit 6B2
TAM: Tamoxifen
ADR: Adriamycin
DP: Docetaxel
AK4: Adenylate kinase 4
PRMT5: Protein arginine methyltransferase 5
HR: Homologous recombination repair
SOX4: SRY box transcription factor 4
JPX: X-inactive specific transcript
PDK1: Phosphatidylinositol dependent kinase 1
EPHB3: Eph receptor B3
NF-κB: Nuclear factor kappa-B
TNFAIP3: Tumor necrosis factor α induced protein 3
VM: Vasculogenic mimicry
CDK12: Cyclin dependent kinase 12
MBNL1: Muscleblind like 1
IRES: Internal ribosome entry site
KHSRP: KH-type splicing regulatory protein
XRN2: 5'-3' Exoribonuclease 2
STAT1: Signal transducer and activator of transcription 1
MDSC: Myeloid derived suppressor cell
CXCL1: Chemokine: C-X-C motif ligand 1
DCs: Dendritic cells
NK: Natural killer
ICI: Immune checkpoint inhibitor
TRIM25: Tripartite motif containing 25
circNDUFB2: Circular RNA NADH dehydrogenase (Ubiquinone) 1 beta subcomplex subunit 2
RIG-I: Retinoic acid inducible gene I
SNHG5: Small nuclear RNA host gene 5
CAFs: Cancer-associated fibroblasts
MAPK: Mitogen-activated protein kinase
NEAT1: Nuclear paraspeckle assembly transcript1
TAMs: Tumor-associated macrophages
DEGs: Differentially expressed genes
TDM2: Type 2 diabetes mellitus
TPO-R: Thrombopoietin receptor
ITP: Immune thrombocytopenia
PHD: Proline-4 hydroxylase
FP: Fluorescence polarization
NRL: Nucleotide recognition lid
GE: Gefitinib
BCRP: Breast cancer resistance protein
MRP-7: Multidrug resistance protein-7
IDH: Isocitrate dehydrogenase
IR: Ionizing radiation
T-ALL: T-cell acute lymphoblastic leukemia
AI: Artificial intelligence
ML: Machine learning
CADD: Computer-aided drug design
DELs: DNA-encoded libraries
